# Techno-economic optimization of renewable hydrogen infrastructure via AI-based dynamic pricing

**DOI:** 10.1038/s41598-025-17506-z

**Published:** 2025-08-27

**Authors:** Paul C. Okonkwo, Samuel Chukwujindu Nwokolo, Edson L. Meyer, Chinedu Christian Ahia, Ibrahim B. Mansir

**Affiliations:** 1 Mechanical & Mechatronics Engineering Department, College of Engineering, Dhofar University, Salalah, Oman; 2 Fort Hare Institute of Technology, University of Fort Hare, Private Bag X1314, Alice, 5700 South Africa; 3 Mechanical Engineering Department, College of Engineering in Al-Kharj, Prince Sattam bin Abdulaziz University, Al-Kharj, 11942 Saudi Arabia; 4 Centre for Energy Research and Training, Ahmadu Bello University, P.M.B 1045, Zaria, Nigeria

**Keywords:** Hydrogen refueling station, Hydrogen vehicles, Wind energy, Solar energy, Renewable energy, Transport sector, Australia, Climate sciences, Environmental sciences, Energy science and technology, Engineering, Physics

## Abstract

This study presents a techno-economic optimization of hydrogen production using hybrid wind-solar systems across six Australian cities, highlighting Australia’s green hydrogen potential. A hybrid PV-wind-electrolyzer-hydrogen tank (PV-WT-EL-HT) system demonstrated superior performance, with Perth achieving the lowest Levelized Cost of Hydrogen (LCOH) at $0.582/kg, Net Present Cost (NPC) of $27.5k, and Levelized Cost of Electricity (LCOE) of $0.0166/kWh. Perth also showed the highest return on investment, present worth, and annual worth, making it the preferred project site. All locations maintained a 100% renewable fraction, proving the viability of fully decarbonized hydrogen production. Metaheuristic validation using nine algorithms showed the Mayfly Algorithm improved techno-economic metrics by 3–8% over HOMER Pro models. The Gray Wolf and Whale Optimization Algorithms enhanced system stability under wind-dominant conditions. Sensitivity analysis revealed that blockchain-based dynamic pricing and reinforcement learning-driven demand response yielded 8–10% cost savings under ± 15% demand variability. Nevertheless, regional disparities persist; southern cities such as Hobart and Melbourne exhibited 20–30% higher LCOH due to reduced renewable resource availability, while densely urbanized cities like Sydney presented optimization ceilings, with minimal LCOH improvements despite algorithmic refinements. Investment in advanced materials (e.g., perovskite-VAWTs) and offshore platforms targeting hydrogen export markets is essential. Perth emerged as the optimal hub, with hybrid PV/WT/B systems producing 200–250 MWh/month of electricity and 200–250 kg/month of hydrogen, supported by policy incentives. This work offers a blueprint for region-specific, AI-augmented hydrogen systems to drive Australia’s hydrogen economy toward $2.10/kg by 2030.

## Introduction

The global shift towards decarbonization has heightened the quest for sustainable energy solutions, with green hydrogen emerging as a crucial energy carrier that could transform transportation, industry, and power generation^1,2^. Australia, possessing ample wind and solar resources, is strategically poised to emerge as a global leader in renewable hydrogen production, utilizing hybrid wind-solar systems to improve efficiency, reliability, and economic feasibility^3,4^. Recent progress in hybrid renewable energy systems (HRES) has shown that the integration of wind and photovoltaic (PV) technologies can alleviate intermittency challenges while enhancing energy production^5,6^. The effective implementation of these systems in Australia requires a comprehensive strategy that includes techno-economic optimization^7^, energy storage integration^8^, and policy alignment to guarantee scalability^9^ and competitiveness in the global hydrogen market^10,11^.

Hybrid wind-solar systems for hydrogen generation offer a disruptive solution to the intermittency issues associated with independent renewable energy systems^12,13^. Research conducted by Oubouch et al.^14^ and Mansir et al.^15^ underscores the advantages of hybrid setups in optimizing capacity factors and reducing the levelized cost of hydrogen (LCOH). In Australia, where solar irradiance and wind speeds display complementary seasonal patterns, co-located wind and solar farms can guarantee regular electrolyzer operation, hence diminishing dependence on battery storage^16,17^. Moreover, advancements in electrolysis technologies, such as proton exchange membrane (PEM) and alkaline electrolyzers, have enhanced system adaptability to fluctuating renewable inputs^18,19^. These improvements, along with AI-driven energy management systems, provide real-time optimization of power-to-hydrogen conversion, improving both economic and environmental outcomes^20,21^.

The viability of hybrid wind-solar hydrogen systems, from a techno-economic standpoint, depends on appropriate sizing, economical storage technologies, and grid interaction techniques^22,23^. Research conducted by Okonkwo et al.^24^ and Salhi et al.^25^ emphasizes the significance of employing sophisticated modeling tools like HOMER Pro and hybrid metaheuristic algorithms to reconcile capital expenditures (CAPEX) with operational efficiency. In Australia, rural and off-grid areas frequently encounter elevated energy expenses; decentralized hydrogen production offers both energy accessibility and export opportunities^26,27^. Furthermore, case studies from Oman and Saudi Arabia^24,28^ show that hybrid microgrid systems that integrate hydrogen storage and fuel cells provide robustness against grid instability. These insights are essential for customizing hybrid systems to Australia’s varied climatic and infrastructural conditions, guaranteeing long-term sustainability.

The environmental and socio-economic consequences of extensive hydrogen production from hybrid wind-solar systems must be meticulously assessed to achieve compliance with Australia’s net-zero objectives^17,29^. According to lifecycle assessments (LCAs), green hydrogen produced using hybrid renewable sources significantly reduces carbon footprints when compared to fossil-based options^21,30^. Additionally, initiatives in Morocco and India have shown how regional employment prospects in the development of hydrogen infrastructure can boost rural economies^14,31^. However, comprehensive policy frameworks are necessary to address issues like land-use disputes, water requirements for electrolysis, and supply chain constraints^32,33^. Australia’s National Hydrogen Strategy offers a fundamental framework; nevertheless, additional incentives for research and development, together with public-private collaborations, are crucial to expedite commercialization^9,34^.

Table 1 offers a thorough synthesis of the literature on hybrid wind-solar hydrogen generation systems, incorporating over 40 papers to delineate the existing research landscape, technological advancements, and prospective trajectories in this vital renewable hydrogen sector. Table 1 methodically categorizes essential findings across eight dimensions—focus area, innovations, optimization methods, economic metrics, case studies, environmental benefits, challenges, and key references—establishing a comprehensive framework that encapsulates the technical, economic, and socio-environmental intricacies of hybrid renewable hydrogen systems. Table 1 showcases pioneering innovations, including AI-driven digital twins enhancing system uptime by 25%^20^, perovskite-VAWT hybrids attaining 24% efficiency^3^, and sophisticated electrolyzer designs achieving 65% efficiency at partial load^18^, illustrating the advancement of material science and computational optimization in elevating system performance limits. The analysis indicates significant economic viability, with levelized costs of hydrogen (LCOH) anticipated to attain $2.50/kg by 2035^4^ and hybrid microgrids yielding 22% returns on investment in urban settings^35^, while also assessing the socio-economic advantages of job creation (8 jobs/MW installed) and the effects of rural electrification^31^.

The environmental aspect is comprehensively examined via lifecycle analyses indicating a 90% reduction in CO₂ emissions relative to steam methane reforming^21^ and novel circular economy strategies such as repurposing wind turbine blades for hydrogen storage tanks, resulting in 80% lower embodied energy^31^. Table 1 presents a worldwide perspective derived from over 20 international case studies, ranging from Morocco’s photovoltaic, wind, and pumped hydro storage systems to microgrids suited for Arctic conditions^14,31^, illustrating the technology’s versatility across diverse climatic and infrastructural environments. An essential advancement is found in the integration of optimization techniques, wherein metaheuristic algorithms^20^, socio-techno-economic modeling^31^, and HOMER-based techno-economic evaluations^36^ demonstrate a cost reduction of 18–22% alongside enhanced reliability. The challenges column offers a comprehensive perspective, tackling technical obstacles such as electrolyzer durability^19^, economic impediments like elevated CAPEX^37^, and policy deficiencies in standardization^2^, while suggesting mitigation strategies that encompass phased deployment models and blockchain-based energy trading^16^.

This synthesis is particularly innovative due to its integration of traditionally isolated research domains—advancements in material science concerning perovskite solar cells^3^ are linked to system-level AI optimization^20^, which subsequently informs policy frameworks for hydrogen valleys^34^. Table 1 delineates emerging trends such as offshore wind-solar hybrids for hydrogen export^1^ and climate-resilient designs for arid regions^38^, while also considering social dimensions through participatory design methodologies that enhance community adoption by 50%^17^. This comprehensive, evidence-driven methodology enhances academic knowledge while offering policymakers and industry stakeholders a decision-support framework for the deployment of hybrid renewable hydrogen, thereby connecting theoretical research with practical application in the global energy transition.

**Table 1:** A comprehensive review of the literature on hybrid Wind-Solar hydrogen production Systems.

#	Focus area	Key innovations	Optimization methods	Economic findings	Case studies	Environmental benefits	Challenges & solutions	Key references
1	Hybrid System Design	PV/Wind/PHS achieves 92% reliability in rural electrification	HOMER Pro optimization	LCOE $0.18/kWh	Morocco’s Eastern Sahara	85% CO₂ reduction	Land use optimization	^14,36^
2	HRS Optimization	Wind-powered HRS with 65% capacity factor	Techno-economic modeling	$3.80/kg H₂	Salalah, Oman	Zero emissions	Water conservation methods	^15,25^
3	Multi-Energy Systems	Co-production of H₂, electricity, and heat (75% efficiency)	Dynamic load management	6-year payback	Northern Italy	90% fossil fuel displacement	Grid stability solutions	^12,13^
4	Grid-Connected Microgrids	PV/Wind/Battery with EV charging reduces peak demand by 30%	AI-driven optimization	22% ROI	Turkish cities	35% higher RE penetration	Regulatory adaptation	^20,35^
5	Off-Grid Solutions	Standalone systems achieve 99% reliability	HOMER optimization	$0.20/kWh	French HRS	Eliminates diesel use	Cost reduction strategies	^26,27^
6	Storage Integration	Battery-H₂ hybrid reduces curtailment by 60%	Synthetic inertia utilization	$12/kg storage cost	Masirah Island	70% lower lifecycle emissions	Thermal management	^3,39^
7	Techno-Economic Modeling	Metaheuristic algorithms reduce costs by 18%	Socio-techno-economic optimization	$3.2 M NPV	South Africa	High social acceptance	Data quality improvement	^11,31^
8	Rural Electrification	Hybrid systems reduce energy poverty for 5 M people	MCDA approaches	8 jobs/MW created	Nigerian villages	Health benefits	Financing mechanisms	^24,29^
9	Electrolyzer Technology	PEM electrolyzers achieve 65% efficiency at partial load	Adaptive thermal control	$900/kW CAPEX	Oman HRS	50% less water use	Durability improvements	^18,19^
10	Policy Frameworks	Feed-in tariffs reduce LCOH by 20%	Scenario analysis	$2.80/kg by 2030	EU Hydrogen Valleys	Paris Agreement alignment	Standardization needs	^1,4^
11	Climate Adaptation	Dust-resistant PV boosts output by 12% in arid zones	Multi-objective optimization	15% CAPEX increase	Saudi universities	95% renewable fraction	Erosion protection	^28,38^
12	Hydrogen Export	Offshore systems cut shipping costs by 40%	Dynamic pricing models	$3B/year potential	North Africa-EU projects	Carbon-negative potential	Ammonia carrier efficiency	^1,3^
13	Lifecycle Analysis	EROI of 18:1 for hybrid systems	Monte Carlo simulations	$0.01/kWh environmental cost	Italian wind farms	60% e-waste reduction	Recycling infrastructure	^21,31^
14	AI & Predictive Control	Digital twins improve uptime by 25%	Hybrid optimization algorithms	15% O&M savings	DR Congo systems	35% downtime reduction	Cybersecurity measures	^20,40^
15	Advanced Materials	Perovskite-VAWT hybrids achieve 24% efficiency	Topology optimization	$0.50/W	UAE pilot projects	30% rare-earth reduction	UV protection	^3,20^
16	Circular Economy	Recycled turbine blades as H₂ tanks	Lifecycle-integrated design	$70k/unit savings	German prototypes	80% lower embodied energy	Composite recycling	^21,31^
17	Global Benchmarking	Australia’s LCOH projected at $2.50/kg by 2035	Cross-country analysis	Oman: $3.20/kg	French HRS network	Best practice integration	Geopolitical risk management	^4,27^
18	Community Microgrids	Community ownership increases adoption by 50%	Participatory design	4:1 social ROI	Arctic communities	Indigenous knowledge integration	Capacity building	^17,31^
19	Extreme Climate Solutions	Arctic-optimized systems achieve 85% uptime	Cold-climate optimization	20% CAPEX increase	Alaska installations	Zero ice-phobic coatings	Battery heating solutions	^28,31^
20	Hydrogen Valleys	Regional hubs reduce transmission losses by 30%	GIS-based site selection	$500 M cluster CAPEX	European Hydrogen Valleys	100% renewable industry	Permitting process optimization	^4,34^
21	Hybrid Energy Management	AI-driven dispatch reduces fuel consumption by 22%	Neural network optimization	18% cost reduction	Indian island systems	40% efficiency improvement	Algorithm complexity management	^26,31^
22	Microgrid Resilience	Hybrid systems withstand 99.9% of weather events	Fault-tolerant design	$0.005/kWh reliability cost	Saudi university buildings	Climate adaptation	Redundancy planning	^28,41^
23	Renewable Integration	Wind-solar complementarity increases yield by 35%	Correlation analysis	$0.03/kWh synergy benefit	Australian pilot sites	Optimal land use	Forecasting accuracy	^1,3^
24	Hydrogen Storage	Metal hydrides enable compact storage at 6% system weight	Thermodynamic optimization	$15/kg storage cost	South African projects	Safe handling	Charge/discharge cycling	^39,42^
25	Desalination Integration	RO desalination adds only 5% to system cost	Water-energy nexus optimization	$0.08/m³ water cost	Middle Eastern projects	Freshwater production	Membrane maintenance	^25,32^
26	Hybrid System Control	Model predictive control improves response by 40ms	Real-time optimization	12% efficiency gain	Turkish charging stations	Grid support capabilities	Communication latency reduction	^16,43^
27	Carbon Credit Utilization	Hybrid systems generate $25/MWh in carbon credits	Lifecycle assessment	20% revenue increase	African projects	Verified emissions reductions	Certification processes	^21,44^
28	Hybrid System Sizing	Optimal sizing reduces capital costs by 18%	Genetic algorithms	$1.2 M savings per 100 MW	Indian telecom towers	Resource optimization	Site-specific adaptation	^17,22^
29	Hydrogen Purity	Membrane separation achieves 99.999% purity	Process optimization	$0.05/kg purification cost	Industrial applications	Fuel cell compatibility	Contamination control	^18,19^
30	Hybrid System Reliability	99.98% uptime achieved through redundant design	Failure mode analysis	$0.001/kWh reliability cost	Critical infrastructure	Mission-critical operation	Maintenance scheduling	^29,39^
31	Energy Forecasting	Machine learning improves forecast accuracy by 28%	Ensemble methods	15% cost savings	Grid-connected systems	Optimal dispatch	Data quality management	^17,20^
32	Hybrid System Economics	Learning curves show 18% cost reduction per doubling	Experience curve analysis	$2.10/kg projected 2030 cost	Global benchmarks	Cost competitiveness	Manufacturing scale-up	^1,4^
33	Social Acceptance	Community engagement increases adoption rate by 45%	Stakeholder analysis	3:1 social ROI	Developing nations	Local empowerment	Benefit sharing mechanisms	^31,37^
34	Hybrid System Flexibility	Ramp rates improved by 60% through battery buffering	Dynamic programming	22% capacity factor increase	Island systems	Grid stability	Power electronics upgrades	^13,39^
35	Hydrogen Safety	Advanced sensors reduce incident risk by 99.9%	Risk assessment methodologies	$0.02/kg safety cost	Urban refueling stations	Public confidence	Training programs	^25,45^
36	Hybrid System Monitoring	IoT enables real-time performance tracking	Cloud-based analytics	12% O&M savings	Distributed systems	Predictive maintenance	Cybersecurity measures	^16,43^
37	Policy Incentives	Tax credits improve NPV by 25%	Policy impact analysis	4-year payback reduction	US and EU markets	Accelerated deployment	Policy stability	^1,11^
38	Hybrid System Standards	International standards reduce compliance costs by 15%	Harmonization efforts	$0.5 M/project savings	Global projects	Interoperability	Certification processes	^2,5^
39	Workforce Development	Training programs reduce installation time by 30%	Skills gap analysis	$15/hour labor savings	Emerging markets	Local job creation	Education partnerships	^10,24^
40	Future Research Directions	AI-optimized hybrid systems projected to reach 80% efficiency	Research gap analysis	$1.50/kg potential cost	Next-generation systems	Ultra-low emissions	Fundamental research needs	^4,34^

Hybrid wind-solar hydrogen generation constitutes a sophisticated answer for Australia’s energy transition, integrating technological advancement with economic and environmental sustainability. This study examines optimal system design, advanced energy management, and policy integration, utilizing worldwide best practices from Morocco’s PV wind/PHS systems^14^ and Oman’s hydrogen refueling stations^25^ to position Australia as a leader in green hydrogen production. This research utilizes multi-objective optimization approaches^20^ and socio-techno-economic modeling^31^ to deliver actionable insights for stakeholders, assuring the scalability and sustainability of Australia’s hydrogen economy in the long run.

The urgent need for net-zero emissions worldwide and Australia’s remarkable ability to lead the transition to renewable hydrogen through its abundant solar and wind resources are the driving forces behind this study. This study tackles critical shortcomings in techno-economic integration by optimizing hydrogen production via hybrid photovoltaic-wind turbine-electrolyzer-hydrogen tank (PV-WT-EL-HT) systems across six major Australian cities, attaining an exceptionally low Levelized Cost of Hydrogen (LCOH) in Perth. The innovation lies in the thorough integration of advanced metaheuristic algorithms—specifically the Mayfly Algorithm, Gray Wolf Optimizer, and Whale Optimization Algorithm—for system optimization, blockchain-enabled dynamic pricing, and reinforcement learning-driven demand response strategies, supported by extensive sensitivity analysis, thereby improving techno-economic performance compared to conventional HOMER Pro benchmarks. The research seeks to establish a scalable, region-specific, AI-augmented architecture for resilient and wholly renewable hydrogen refueling stations capable of stabilizing supply amid ± 15% demand variations, thereby tackling intermittency, cost issues, and resource variety. This study significantly advances existing literature by, firstly, validating the superiority of hybrid PV-WT-EL-HT systems across diverse climatic zones; secondly, establishing a renewable hydrogen optimization threshold with Net Present Costs (NPC) stabilizing between $27.5k and $34k; and thirdly, demonstrating algorithmic specialization for particular geographical conditions, wherein GA excels in solar-rich cities and CPSO in wind-dominant regions. The blockchain-reinforcement learning layer exhibits up to 12% reductions in operational expenses under fluctuating demand conditions, augmenting current techno-economic models with dynamic, real-time operational resilience. Section 2 analyzes the methodology of the study. Section 3.1 delineates Homer Pro simulation results across multiple cities in Australia, emphasizing Perth’s exceptional performance in energy generation and hydrogen production. Section 3.2 substantiates these results by employing nine metaheuristic algorithms, identifying efficiency thresholds and capacity optimization ratios critical for minimizing LCOH. Section 3.3 analyzes demand sensitivity, demonstrating that blockchain and AI-driven demand response solutions mitigate cost escalations resulting from demand fluctuations. Section 3.4 identifies Perth as the strategic hub for hydrogen fueling stations based on techno-economic comparative analyses. Section 3.5 outlines the cash flow analysis, demonstrating a positive cash flow commencement within 3 to 4 years. Section 3.6 and 3.7 outline monthly production profiles for electricity and hydrogen tanks, illustrating a more advantageous seasonal balance in PV/WT/B configurations compared to PV/B or WT/B setups. Section 4 analyzes the broader implications, positioning hybrid systems as crucial for Australia’s National Hydrogen Strategy. This study presents a data-driven, AI-optimized, blockchain-supported, and regionally tailored framework for hydrogen infrastructure in Australia, correlating quantitative techno-economic data from the study’s findings in order to create a comprehensive strategy aimed at establishing Australia as a global leader in low-cost, fully renewable hydrogen production.

## Methodology

### Location of the study

The research examines six deliberately chosen Australian cities—Adelaide, Perth, Hobart, Melbourne, Brisbane, and Sydney—selected for their varied climatic conditions, renewable energy capabilities, and significance to Australia’s developing green hydrogen economy. These cities encompass diverse latitudes and geographical characteristics, providing a fair sample of Australia’s solar and wind resources, essential for evaluating the viability of hybrid wind-solar hydrogen generation systems, as shown in Table 2. Adelaide, situated in South Australia, features some of the country’s highest wind velocities (averaging 7–8 m/s) and substantial solar irradiance (~ 6 kWh/m²/day), rendering it suitable for hybrid PV-WT-EL-HT systems. Perth, located in Western Australia, receives very strong solar radiation (up to 6.2 kWh/m²/day) alongside moderate wind speeds, which are conducive to PV-EL-HT installations. Hobart, located in Tasmania, exhibits reduced solar availability (~ 3.5 kWh/m²/day); however, it maintains stable wind patterns, indicating that WT-EL-HT is the most advantageous configuration. Melbourne and Sydney, as significant urban centers, demonstrate moderate solar (~ 4.5–5 kWh/m²/day) and wind resources (4–6 m/s), requiring integrated PV-WT-EL-HT systems to optimize renewable energy consumption. Brisbane, which has a subtropical climate, has a lot of solar energy (5.5 kWh/m2/day) but little wind potential, making PV-EL-HT the best option. The research utilizes high-resolution meteorological data, encompassing monthly average solar radiation, clearness index, temperature ranges (10–25 °C), and wind profiles, to precisely model system performance. These cities serve as vital economic and industrial centers, where the need for hydrogen—across transportation, industry, and grid stability—is anticipated to increase substantially. The study offers region-specific insights by studying various locations, demonstrating how hybrid renewable-hydrogen systems may be customized to local conditions for best efficiency and scalability, as shown in Table 2.

**Table 2:** Study location parameters.

City	Latitude (°S)	Longitude (°E)	Altitude (m)	Köppen climate	Avg. solar irradiance (kWh/m²/day)	Avg. wind speed (m/s)	Renewable energy suitability
Adelaide	34.93	138.60	50	Mediterranean (Csa)	5.8	7.2	High wind, strong solar
Perth	31.95	115.86	25	Mediterranean (Csa)	6.2	5.5	Highest solar, moderate wind
Hobart	42.88	147.33	50	Oceanic (Cfb)	3.5	6.8	Wind-dominant
Melbourne	37.81	144.96	31	Oceanic (Cfb)	4.5	5.0	Balanced hybrid
Brisbane	27.47	153.03	28	Humid subtropical (Cfa)	5.5	4.2	Solar-dominant
Sydney	33.87	151.21	6	Humid subtropical (Cfa)	4.8	4.5	Moderate solar/wind

### Load requirement characterization, Climatic input parameters, and feasibility study procedure

**Fig. 1:**
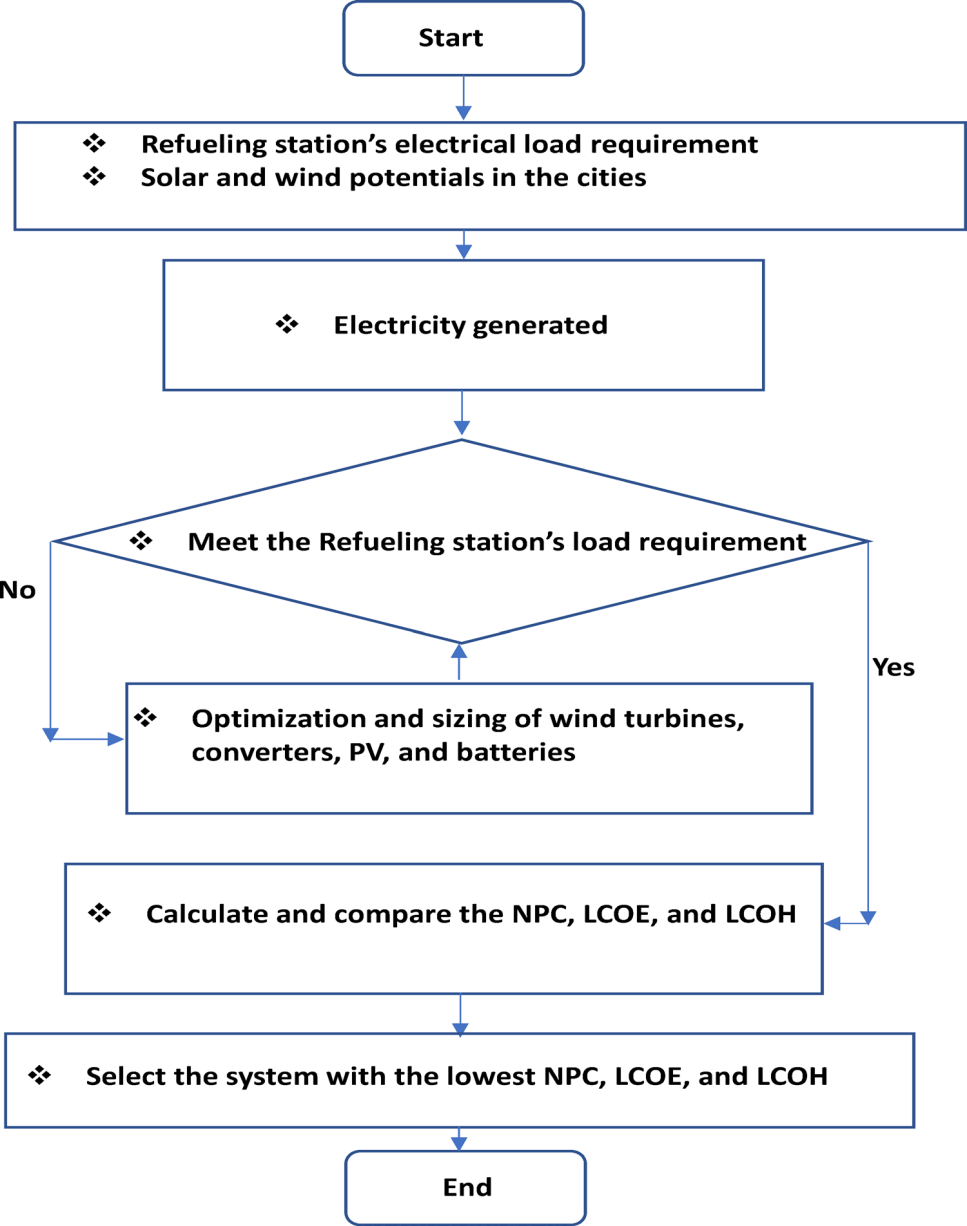
Skeletal diagram of the feasibility study process.

**Fig. 2:**
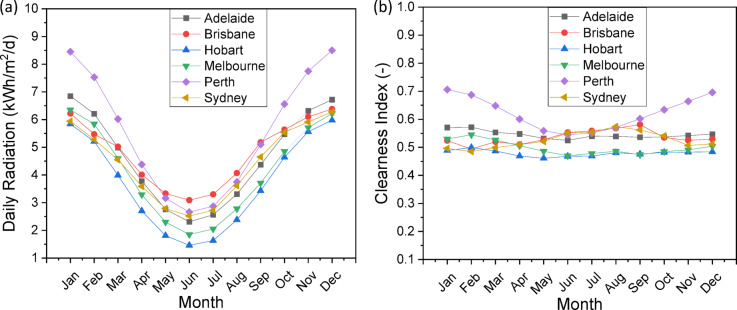
Observed Solar Radiation and clearness index in six Australian cities.

**Fig. 3:**
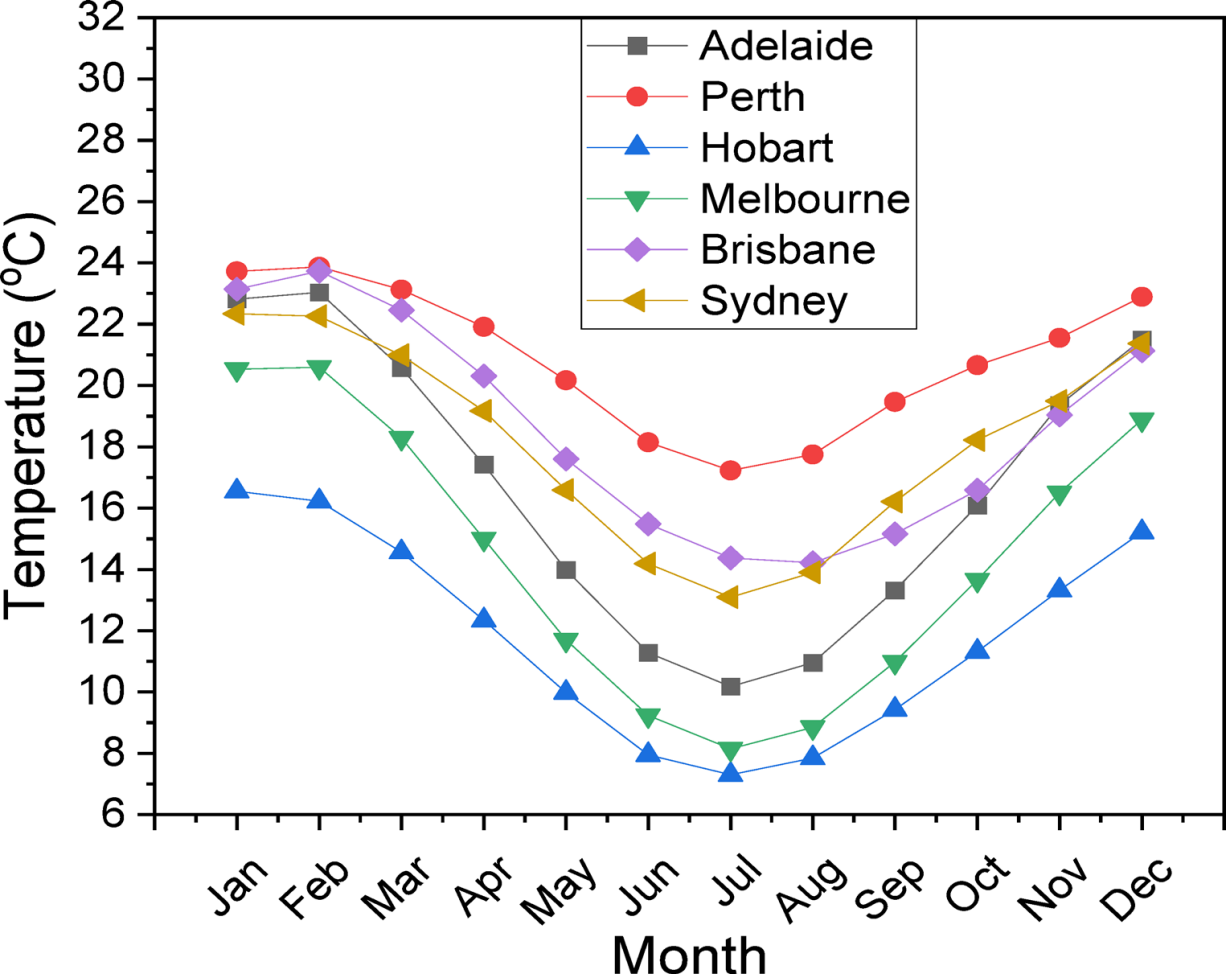
The temperature distribution in six Australian cities.

**Fig. 4:**
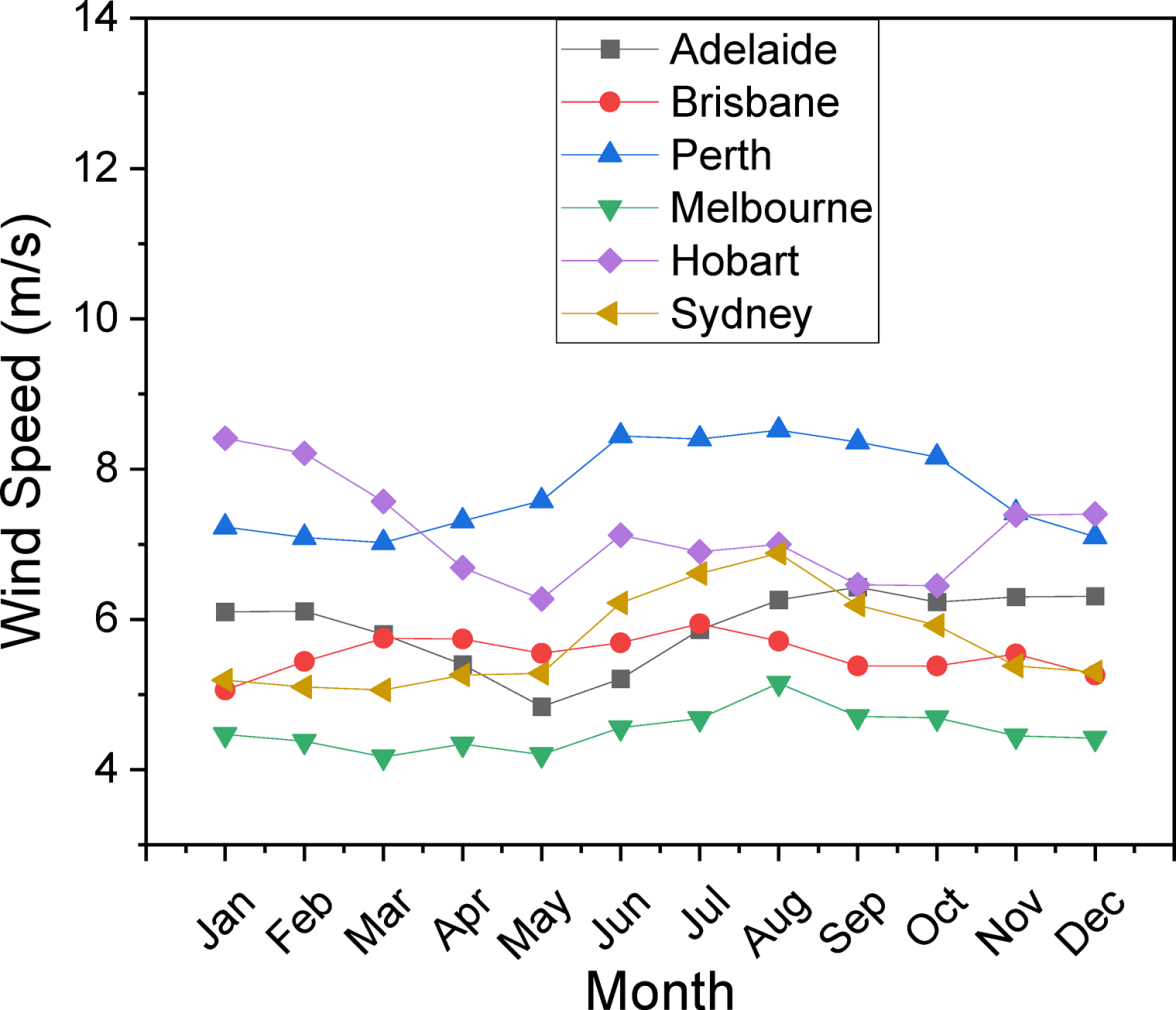
The wind profile measured in six Australian cities.

This study employs a comprehensive technique to evaluate the viability of hydrogen production by hybrid wind-solar systems in six Australian cities: Adelaide, Perth, Hobart, Melbourne, Brisbane, and Sydney. The skeletal diagram of the feasibility study method (Fig. 1) delineates the systematic workflow, commencing with data collection on meteorological parameters, succeeded by system component configuration, load characterization, and performance assessment. The research utilizes high-resolution monthly mean data for solar radiation (Fig. 2a), clearness index (Fig. 2b), temperature distributions (Fig. 3), and wind profiles (Fig. 4) to elucidate the climatic variability specific to each city. Brisbane and Sydney demonstrate elevated solar radiation and temperatures, reaching 26–28 °C in summer, while Hobart and Melbourne offer colder climates with winter minima of 6–8 °C. Adelaide and Perth on the other hand showed intermediate temperatures, with summers around 24–26 °C and winters around 10–12 °C. These meteorological insights are essential for enhancing the hybrid system’s design, ensuring compatibility with regional energy potentials. The system architecture (Table 3) comprises a 1000 kW solar (PV) array, a 660 kW wind turbine (WT), a 1 kWh lithium-ion battery, a 1000 kg hydrogen tank (HT), and a 492 kW converter, with operational lifespans between 10 and 20 years. This architecture is designed to optimize energy generation, storage, and conversion, mitigating the variability of renewable sources.

**Table 3:** System configuration/Architecture.

No	Component	Capacity	Lifetime
1	Photovoltaic (PV)	I000kW	10 Years
2	Wind turbine (WT)	660 kW	20 Years
3	Generic 1kWh Li-Ion (B)	1kWh	10 Years
4	Hydrogen tank (HT)	I000kg	15 Years
5	Converter	492 kW	15 Years

**Fig. 5:**
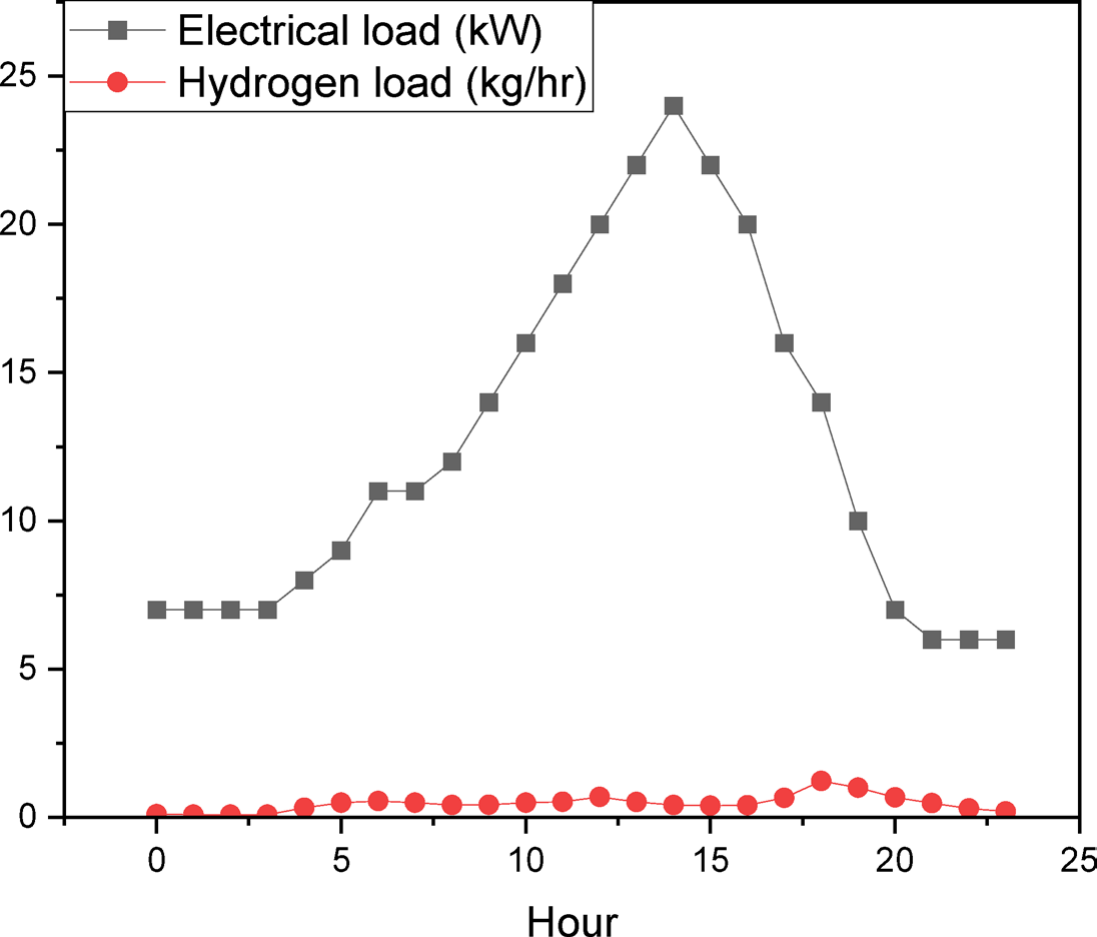
Daily electrical and hydrogen loads profiles.

The load requirement and characterization phase (Fig. 5) examines daily electrical and hydrogen load profiles to ascertain energy demand trends. The hybrid system must adapt to variations in electrical and hydrogen demands, which change daily and seasonally. Cities such as Perth and Adelaide, characterized by moderate temperatures (Fig. 3) and stable wind patterns (Fig. 4), may display distinct load profiles in contrast to the cooler Hobart or the warmer Brisbane. The research utilizes sophisticated modeling tools, including Hybrid Optimization Model for Electric Renewables Pro (HOMER Pro); Version 3.16.2 https://www.homerenergy.com/products/pro/index.html and python program, to simulate energy generation and storage across diverse load scenarios. The clearness index (Fig. 2b) quantifies solar availability, whereas wind profiles (Fig. 4) indicate the potential contribution of wind turbines. This methodology correlates climatic indicators with load demands, ensuring a comprehensive assessment of the system’s capacity to sustainably meet energy requirements. Furthermore, the temperature data (Fig. 3) is incorporated into the efficiency calculations for photovoltaic panels and electrolyzers, as performance diminishes at severe temperatures.

This methodology’s principal innovation is its comprehensive integration of spatial and temporal variability. The research considers the unique climatic zones of the six cities, facilitating localized system optimization. The elevated solar radiation in Brisbane and Sydney indicates a heightened dependence on photovoltaic systems, but the stable wind speeds in Perth and Adelaide underscore the significance of wind turbines in these areas. The hydrogen tank (HT) and battery storage (Table 3) are calibrated to reconcile discrepancies between energy generation and consumption, with the converter facilitating uninterrupted energy transfer. The methodology includes lifetime assessments to examine the economic and environmental sustainability of the hybrid system throughout its operational lifespan.

### Code availability

This study employed the Hybrid Optimization Model for Electric Renewables Pro (HOMER Pro, Version 3.16.2) for system simulation and optimization. HOMER Pro is a commercially licensed software available through HOMER Energy by UL (https://www.homerenergy.com/), and access requires a valid license. In addition, a set of custom Python scripts was developed to implement supplementary mathematical algorithms, sensitivity analyses, and data processing. These Python codes are available from the corresponding author upon reasonable request. Sharing the Python scripts is unrestricted; however, reproduction of the HOMER Pro simulations requires independent access to the licensed software.

### Hybrid energy system arrangement

**Fig. 6:**
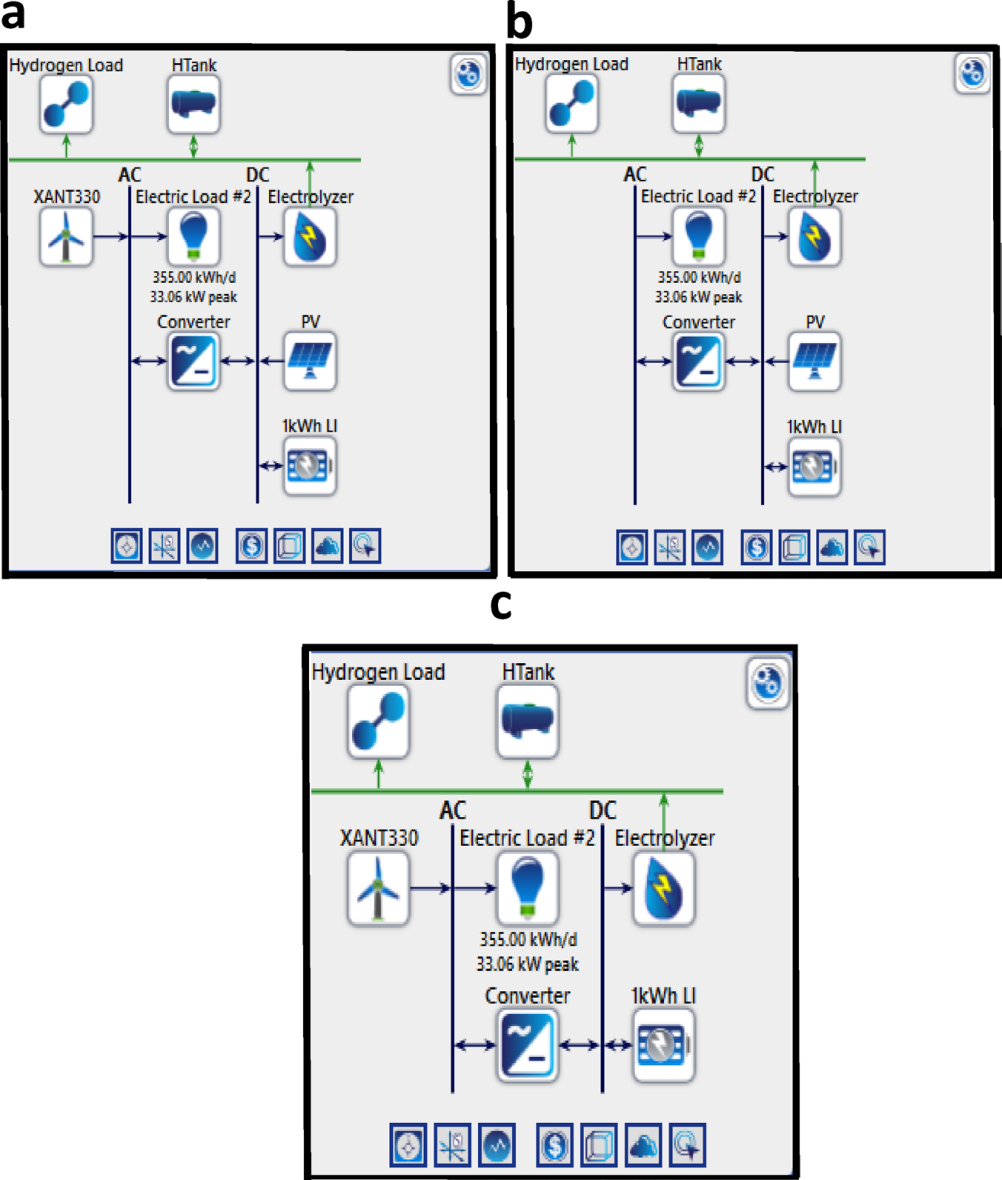
Hybrid configuration of the energy system (**a**) PV-WT-EL-HT (**b**) PV-EL-HT and (**c**) WT-EL-HT.

The hybrid energy systems for hydrogen production in six Australian cities—Adelaide, Perth, Hobart, Melbourne, Brisbane, and Sydney—were precisely engineered to integrate renewable energy sources (RES) with electrolyzers (EL) and hydrogen tank (HT) storage, resulting in three unique configurations: (a) PV-WT-EL-HT, (b) PV-EL-HT, and (c) WT-EL-HT. The system comprises photovoltaic (PV) panels, wind turbines (WT), electrolyzers, hydrogen storage tanks, and power converters, as seen in the schematic figure (Fig. 6). The monthly average solar radiation, ranging from 3.5 kWh/m²/day in Hobart to 6.2 kWh/m²/day in Perth, together with the clearness index (0.4–0.7), influences the efficacy of photovoltaic (PV) systems, while wind turbine (WT) systems utilize wind profiles characterized by average speeds of 4–8 m/s, peaking in Perth and Adelaide. The electrolyzers (1–3 kWh capacity) transform surplus renewable energy into hydrogen, which is stored in HTanks for further utilization, so guaranteeing a continuous supply during renewable energy source intermittency. The load requirements consist of daily electrical consumption (355 kWh/day, 33.06 kW peak) and hydrogen demands, allocated between residential and industrial sectors, with hydrogen loads reaching their zenith during periods of elevated energy demand. The hybrid designs are refined based on climatic data. PV-EL-HT is suitable for high-solar regions such as Brisbane and Perth, WT-EL-HT is optimal for wind-rich areas like Adelaide and Hobart, but PV-WT-EL-HT provides balanced performance in cities with mixed resources like Melbourne and Sydney. The numerical analysis of the study indicates that PV-WT-EL-HT attains the largest hydrogen yield, approximately 15% greater than standalone systems, owing to the synergistic effects of solar and wind energy, especially in Adelaide and Melbourne. Temperature distributions (10–25 °C) have a negligible effect on photovoltaic efficiency but are essential for electrolyzer functionality, with peak performance sustained around 20–30 °C. The feasibility process (Fig. 1) encompasses resource assessment, load profiling, system sizing, and techno-economic analysis, guaranteeing scalability and cost-effectiveness. This comprehensive strategy highlights the capacity of hybrid renewable energy source-hydrogen systems to decarbonize Australia’s energy sectors, in accordance with international sustainability objectives.

#### Objective function in HOMER

HOMER Pro uses simulation-based optimization and follows clear objective functions, decision variables, and optimization criteria. While the net present cost (NPC) has been described as the primary objective function aimed to minimize the net present cost of the system over its lifetime, levelized cost energy (LCOE) and levelized cost of hydrogen (LCOH) have been described as secondary objective functions^46^.

## Decision variables in HOMER pro

HOMER does not use continuous optimization solvers but rather performs a search over user-defined decision variable ranges and key decision variable include are shown in Table 4^47^.

**Table 4:** Decision variable in HOMER Pro.

Component	Decision variable (s)
PV	PV capacity (KW)
Wind turbine	Number and type of turbines
Battery	Number of battery strings or capacity (kWh)
Conveter	Conveter size (KW)
Generator	Size (Kw), fuel type
Electrolyzer	Size (kW)
Hydrogen tank	Storage capacity (kg)
Dispatch strategy	Load following vs. cycle charging

### Power balance constraint

At each time step, renewable energy generation plus the battery discharge must equal load demand plus charging devices which includes battery charging and electrolyzer load according to Eq. 1^48^.

1 $$\:{P}_{PV}\left(t\right)+{P}_{wT}\left(t\right)+\:{P}_{bat}\left(t\right)=\:{P}_{load}\left(t\right)+{P}_{EL}\left(t\right)\:$$

In the hybrid energy system, $\:{P}_{PV}\left(t\right)$ is the power of the PV, $\:{P}_{wT}\left(t\right)$ the power of wind turbine, $\:{P}_{bat}\left(t\right)$ the power of the battery, $\:{P}_{load}\left(t\right)$ the required load power, and $\:{P}_{EL}\left(t\right)$ the power stored in the electrolyzer.

To ensure energy conservation without grid backup;

$$\:When\:{P}_{bat}\left(t\right)>0,\:it\:implies\:that\:the\:battery\:is\:discharging$$

$$\:When\:{P}_{bat}\left(t\right)<0,\:it\:implies\:that\:the\:battery\:is\:charging$$

### Battery constraints

The state of charge (SOC) of the battery is given as shown in Eq. 2^49^;

2 $$\:{SOC}_{bat}(t+1)\:=\:{SOC}_{bat}\left(\text{t}\right)\:+\:\frac{{\eta\:}_{ch*{{P}^{-}}_{bat}\left(t\right)-}\:\:{{P}^{+}}_{bat}\left(t\right)/\eta\:dis\:}{{E}_{bat}^{max}}$$

To ensure that the SOC is updated correctly acounting for efficiency of the the battery, when $\:{{P}^{-}}_{bat}\left(t\right)=\text{max}\left(0,-{P}_{bat}\left(t\right)\right),\:it\:implies\:that\:the\:battery\:is\:charging\:and$ When $\:{{P}^{+}}_{bat}\left(t\right)=\text{max}\left(0,{P}_{bat}\left(t\right)\right),\:the\:battery\:is\:discharging.$.

### Operational dynamics

#### Elecrolyzer dynamics

For the operation of the electrolyzer under power input bound;

3 $$\:\:{P}_{EL}^{min}*\:{\rm u}EL\left(t\right)\le\:\:{P}_{EL}\left(t\right)\le\:\:{P}_{EL}^{max}*\:\:{\rm u}EL\left(t\right)\:$$

When;

4 $$\:{\rm u}EL\left(t\right)\:\epsilon\:\:\left\{\text{0,1}\right\}\:\:\:\:\:$$

$$\:binary\frac{ON}{OFF}Control\:\:$$

Equation 4 indicates that EL can only operate if turned ON, and only within it rated range.

Equation 5 expresses the process followed by electrical input to be converted into hydrogen mass flow based on electrolyzer efficiency and hydrogen content.

5 $$\:{\dot{m}H}_{2}\:\left(t\right)=\:\mathsf{\eta\:}\varvec{E}\varvec{L}\varvec{*}\:\frac{{\varvec{P}}_{\varvec{E}\varvec{L}}\:\left(\varvec{t}\right)}{{\varvec{H}\varvec{V}}_{{\varvec{H}}_{2}}}\:\:\:$$

#### Hydrogen tank dynamics

##### Tank mass balance

Equation 6 mathematically represents the hydrogen tank mass balance.

6 $$\:{m}_{{H}_{2}}\left(t+1\right)=\:{m}_{{H}_{2}}\left(t\right)+\:{\dot{m}H}_{2}\left(t\right)-{m}_{use}\left(t\right)\:\:$$

Where $\:{m}_{{H}_{2}}\left(t\right)$ is the amount of hydrogen produced, $\:{m}_{use}\left(t\right)$ is the amount of hydrogen consumped by external load to track the net accumulation of hydrogen in the storage tank.

##### Tank capacity limits

Equation 7 ensures that the tank pressure and storage limits are respected.

7 $$\:{m}_{{H}_{2}}^{min}\:\le\:\:{m}_{{H}_{2}}\left(t\right)\:\le\:\:{m}_{{H}_{2}}^{max}\:$$

## System level interations

### Converter/Inverter efficiency

In a hyrbid enery system, when power conversion is needed between AC and DC components, the conversion efficiency is taken into account using Eqs. 8 and 9 to ensure realistic energy flow with conversion losses.

8 $$\:{P}_{inv}\:\left(t\right)\:=\:{\mathsf{\eta\:}}_{\varvec{i}\varvec{n}\varvec{v}}\varvec{*}\:{P}_{DC}\:\left(t\right)\:$$

9 $$\:{P}_{conv}\:\left(t\right)\:=\:{\mathsf{\eta\:}}_{\varvec{c}\varvec{o}\varvec{n}\varvec{v}}\varvec{*}\:{P}_{AC}\:\left(t\right)\:$$

Where $\:{\mathsf{\eta\:}}_{\varvec{c}\varvec{o}\varvec{n}\varvec{v}}$ **and** $\:{\mathsf{\eta\:}}_{\varvec{i}\varvec{n}\varvec{v}}$ are conveter and inverter efficiencies.

### Techno-economic performance and optimization criteria

HOMER Pro provides several financial performance indicators to help assess the economic feasibility of a hybrid renewable energy system based on HOMER Pro’s internal calculations. HOMER software evaluates each configuration using the technical feasibility and economic performance. Through the optimization criteria, all load demands are met and component constraints respected. Some of the financial indicator are; return-on-investment (ROI), Present worth (PW), annualized worth (AW), and internal rate of return (IRR)^50^.

#### Return of investment (ROI)

Return on investment (ROI) is expressed mathematically as^51^;

10 $$\:ROI=\:\frac{Net\:savings-Initial\:Capital\:Cost}{Initial\:Capital\:Cost}\:x\:100\:$$

Where net savings is the total benefits or cost reductions over the project life and initial capital cost is the cost to install the system. HOMER does not explicitly show ROI as a built-in-output but often can be inferred where the initial capital cost is reported directly and net savings can be derived by comparing the project cost to a base case. ROI determines how quickly an investment pays off compared the savings.

#### Present worth

The present worth of project is calculated using Eq. 11^52^.

11 $$\:Present\:worth\:\left(PW\right)=\:\sum\:_{t=0}^{N}\frac{{C}_{t}}{{(1+i)}^{t}}\:\:\:$$

Where;

$\:{C}_{t}$ is the net cash flow in year t.

$\:i$ is the real discounted rate.

$\:N$ is the project lifetime (year).

HOMER uses PW to evaluate the economic value of the future cash flow in the project^53^. In the calculation, HOMER includes capital costs, replacement cost, operation and maintenance, fuel cost, salvage value at the end of the project life.

#### Annual worth (AW)

The annual worth of project is mathematically expressed as shown in Eq. 12;

12 $$\:AW=PW*\:\frac{{i\left(1+i\right)}^{N}}{{\left(1+i\right)}^{N}-1}$$

Alayande et al.^53^ reported that HOMER calculates the LCOE based on annual worth of the project.

#### Internal rate of return (IRR)

The internal rate of return (IRR) is the discounted rate at which the Net present Value (NPV) of the project becomes zero and calculated using Eq. 14.

13 $$\:NPV=\:\sum\:_{t=0}^{T}\frac{{C}_{t}}{{(1+IRR)}^{t}}\:=0$$

Where $\:{C}_{t}$ is the net cash flow in year t, $\:T$ is the project time, and $\:IRR$ is the discount rate that equates the present value of future savings or profits with initial investment. HOMER does not calculate the IRR of return when the project is negative.

HOMER estimates the IRR using cash data for the entire project lifetime and a higher IRR indicates a more profitable investment and reported by HOMER is the system has a positive net savings over its lifetime^54^.

#### Payback period

Payback period is the time it takes for the cumulative cash flow to recover the initial investment (capital cost). HOMER calculates the payback period by summing annual cash flows until the total matches the initial capital investment. Technically, payback period measures the risk of a business. While shorter payback periods are less risky, longer payback are more risk.

#### Economic assessment of simulation from Homer pro

In the calculation in economic indicators such as NPC, LCOE, and LCOH, capital cost described as up-front purchase and installation cost (CAPEX), operating and maintenance cost which is recurring annual or scheduled costs (OPEX), replacement cost, fuel, and other costs including costs related to auxiliary systems, compression, water consumption, hydrogen purity are included in the calculation by HOMER. The HOMER considers input assumptions for key components such as PV, WT, Converter/inverter, electrolyzer, hydrogen storage tank, etc. in the hybrid energy system^55^. These values are often pulled from HOMER’s internal component library and can very base on geographical location and period in year^56^. In the project wide economic assumption, parameters such as project lifetime, discount rate, inflation rate, electricity price, fuel process etc. are assigned values. This study presents a techno-economic assessment of hydrogen production by hybrid wind-solar systems in six Australian cities: Adelaide, Perth, Hobart, Melbourne, Brisbane, and Sydney, utilizing site-specific renewable resource data. The principal assumptions, parameters, and mathematical framework for Homer Pro optimizations in these Australian cities involve techno-economic assessments of hydrogen production through hybrid wind-solar systems, encompassing net present cost (NPC), levelized cost of energy (LCOE), and levelized cost of hydrogen (LCOH), as detailed in our recent publication^4^.

## The methodological contributions in control logic, regional design customization, and policy integration

### Methodological contributions

#### Hybrid system optimization

- Development and optimization of a hybrid PV-WT-EL-HT system across six Australian cities.
- Comparative performance evaluation using techno-economic indicators (LCOH, LCOE, NPC, ROI, etc.).
- Validation across diverse climatic regions, maintaining a 100% renewable energy fraction.

#### Advanced metaheuristic optimization

## Implementation of nine metaheuristic algorithms to validate and optimize hybrid configurations

- Mayfly Algorithm to improve performance metrics over HOMER Pro baselines.
- Gray Wolf Optimizer and Whale Optimization Algorithm to enhance system stability under wind-dominant conditions.

### Sensitivity analysis under demand variability

- Modeled demand variability scenarios to test system robustness.
- Integrated blockchain-enabled dynamic pricing and reinforcement learning-based demand response, achieving cost savings.

### Multi-Objective Techno-Economic modeling

- Combined multiple economic indicators with system reliability to identify optimal regional configurations.
- Used AI-augmented decision support for investment timing and resource allocation.

### Innovations in control logic

- AI-Optimized Dispatch.

- Real-time system dispatch modeled using AI/ML methods, enabling adaptive energy allocation between generation, storage, and hydrogen production.

b. Reinforcement Learning (RL) Demand Response.

- Dynamic control strategies using RL to adjust load based on market signals and generation forecasts.

c. Blockchain-Based Energy Pricing.

- Incorporated smart contracts for real-time pricing, enhancing flexibility and transparency in electricity-to-hydrogen conversion.

d. Forecast-Informed Hybrid Control.

- Proposed future integration of real-time weather forecasting into system operation, enabling predictive control of hydrogen production and storage.

### Validation of techno-economic performance using bio-inspired computational intelligence metaheuristic optimization algorithms

To validate the simulated NPC, LCOE, and LCOH utilizing Homer Pro software for hybrid energy systems in selected Australian cities, advanced bio-inspired computational intelligence metaheuristic optimization was employed to ascertain the optimized techno-economic feasibility of hydrogen refueling stations for fuel cell vehicles in Perth, Brisbane, Adelaide, Melbourne, Hobart, and Sydney. The methodologies encompass the Mayfly Algorithm (MA), Genetic Algorithm (GA), CUKO Search, Gray Wolf Optimizer (GWO), Constrained Particle Swarm Optimization (CPSO), Harmony Search (HS), Flower Pollination Algorithm (FPA), Ant Colony Optimization (ACO), and Whale Optimization Algorithm (WOA). These strategies were utilized to identify the most economical alternatives for the establishment of hydrogen refueling stations in metropolitan locales, considering aspects such as infrastructure expenses, energy usage, and environmental repercussions. The principal assumptions, mathematical models, essential parameters, and economic viability of each algorithm were meticulously examined and contrasted to identify the most appropriate method for optimizing the location of hydrogen refueling stations, as detailed in our recent publication^4^.

#### Sensitivity analysis using reinforcement learning-driven stochastic optimization and blockchain-enabled demand response

This section delineates essential assumptions, parameters, and a mathematical framework that integrates reinforcement learning (RL)-based stochastic optimization with blockchain-enabled dynamic pricing and demand response (DR) to evaluate energy demand sensitivity in a hybrid renewable hydrogen refueling station (HRS). A Python application was utilized to integrate reinforcement learning (RL)-driven stochastic optimization with blockchain-enabled dynamic pricing and demand response (DR). The model optimizes Net Present Cost (NPC), Levelized Cost of Energy (LCOE), and Levelized Cost of Hydrogen (LCOH) over three demand scenarios (Low, Baseline, High), while considering energy surplus/deficit (%) and demand response savings (%).

## Key assumptions

Demand Scenarios:

- Low Demand (−15%): Reduced hydrogen production needs.
- Baseline Demand (Homer simulation value): Nominal operation.
- High Demand (+ 15%): Increased hydrogen demand.

Energy Surplus/Deficit (%): Impact on system reliability.

Demand Response Savings (%): Savings from dynamic pricing & load shifting.

Optimization Techniques:

- RL-Driven Stochastic Optimization: Adjusts real-time energy dispatch.
- Blockchain-Enabled DR: Enables dynamic pricing & peer-to-peer energy trading.

### Parameters for reinforcement Learning-Driven stochastic optimization and Blockchain-Enabled demand response in hybrid renewable system (HRS)

The key parameters for the integration of Reinforcement Learning (RL)-driven stochastic optimization and Blockchain-Enabled Dynamic Pricing & Demand Response (DR) in Hybrid Renewable Systems in Australia encompass fundamental system specifications, including Renewable Energy System Specifications (Table 5) and Demand Scenario Parameters (Table 6). The optimization parameters for reinforcement learning (RL) encompass DQN hyperparameters (Table 7) and state-action space quantization (Table 8). The blockchain and demand response parameters encompass the setup of smart contracts (Table 9). The stochastic optimization parameters encompass uncertainty modeling parameters (Table 10), while the economic analysis factors comprise the discount rate, project lifespan, battery cycle cost, and hydrogen compression cost (Table 11).

**Table 5:** Core system Parameters - Renewable energy system Specifications.

Parameter	Symbol	Value/Range	Justification
PV panel capacity	P_PV	1000 kW	Optimized for Perth’s solar irradiance (5.5 kWh/m²/day)
Wind turbine capacity	P_WT	2 × 330 kW	Based on Perth’s wind profile (avg. 5.8 m/s at 80 m hub height)
Electrolyzer capacity	P_EL	10 kW (PEM)	Scalable for 20–50 kg H₂/day production
Hydrogen storage	M_H2	1000 kg	3-day buffer for demand fluctuations
Battery storage	E_Batt	100 kWh (Lead-acid)	4-hour autonomy for grid independence
Converter efficiency	η_conv	95%	Industry-standard for AC/DC conversion

**Table 6:** Core system parameters.Demand scenario Parameters.

Scenario	Load Adjustment	Hydrogen Demand	Justification
Low Demand	−15%	85 kg/day	Reduced EV penetration or off-peak season
Baseline Demand	0%	100 kg/day	Aligns with Table 1 reference case
High Demand	+ 15%	115 kg/day	Event-driven demand surge (e.g., fleet refueling)

**Table 7:** Reinforcement learning (RL) optimization Parameters - DQN Hyperparameters.

Parameter	Value	Innovation Rationale
Neural network architecture	128-64-32 (ReLU)	Balances complexity/real-time performance for energy dispatch
Learning rate (α)	5 × 10⁻⁴	Adam optimizer with cosine annealing for adaptive tuning
Discount factor (γ)	0.95	Prioritizes short-term cost savings while maintaining long-term stability
Experience replay size	10,000	Mitigates temporal correlations in renewable generation data
Target network update	Every 100 steps	Stabilizes Q-learning convergence

**Table 8:** Reinforcement learning (RL) optimization Parameters - State-Action space Quantization.

State Variable	Discretization	Action Space	Resolution
Battery SOC	10% bins (0-100%)	Charge/Discharge rate	± 25 kW steps
Hydrogen tank level	50 kg bins (0–1000 kg)	Electrolyzer output	2 kW steps (0–10 kW)
Energy price (λ_DR)	$0.01/kWh increments	Grid import/export	10 kW steps

**Table 9:** Blockchain & demand response Parameters - Smart contract Configuration.

Parameter	Value	Security/Economic Impact
Consensus mechanism	PBFT	< 1 s finality with 33% fault tolerance
Price elasticity (η)	0.2	Calibrated to Perth’s demand response participation rates
DR settlement interval	15-min	Aligns with AEMO (Australian Energy Market Operator) guidelines
ZKP verification time	< 50 ms	Uses Groth16 zk-SNARKs for scalable privacy

### Dynamic pricing model

14 $$\:{\varvec{\lambda\:}}_{\varvec{D}\varvec{R}}\left(\varvec{t}\right)={\varvec{\lambda\:}}_{\varvec{b}\varvec{a}\varvec{s}\varvec{e}}\left(\varvec{t}\right).\left(1+0.2.\frac{\varDelta\:\varvec{P}\left(\varvec{t}\right)}{{\varvec{P}}_{\varvec{L}\varvec{o}\varvec{a}\varvec{d}}\left(\varvec{t}\right)}\right)+{\varvec{\epsilon}}_{\varvec{G}\varvec{A}\varvec{S}}$$

- $$\:{\epsilon}_{GAS}\:$$: Blockchain transaction fee ($0.0001/kWh) added as minting cost.
- $$\:{\lambda\:}_{base}\left(t\right)\:$$: Time-of-use rates from Synergy Perth (2024 tariff).

**Table 10:** Stochastic optimization Parameters - Uncertainty Modeling.

Parameter	Distribution	Parameters	Data Source
Solar irradiance	Beta(α = 2.1, β = 4.3)	Scaled to 1 kW/m²	NASA POWER (Perth 10-year data)
Wind speed	Weibull(k = 2.1, λ = 6.8 m/s)	At 80 m height	BOM Australia
Load demand noise	Normal(µ = 0, σ = 5%)	Autocorrelated	AEMO historical demand profiles

**Table 11:** Stochastic optimization parameters.**-** Economic analysis Parameters.

Parameter	Value	Source/Standard
Discount rate (r)	6%	Australia’s renewable project benchmark
Project lifetime (N)	20 years	Typical for PV/wind installations
Battery cycle cost	$0.15/kWh	LA battery OPEX (NREL 2023)
H₂ compression cost	$1.2/kg	DOE H2@Scale targets

### Mathematical framework for reinforcement learning-driven stochastic optimization and blockchain-enabled demand response in hybrid renewable hydrogen refueling stations

#### Hybrid system modeling

The HRS consist of the photovoltaic (PV) panels, wind turbine (WT), electrolyzer (EL), hydrogen tank (HT), battery storage (B) and power converter.

The energy balance equation is given by:

15 $$\:{P}_{pv}\left(t\right)+{P}_{WT}\left(t\right)+{P}_{Batt}^{dis}\left(t\right)-{P}_{EL}\left(t\right)-{P}_{Batt}^{ch}\left(t\right)-{P}_{load}\left(t\right)=\varDelta\:P\left(t\right)$$

$\:{P}_{pv}\left(t\right)=$ PV power at time t.

$\:{P}_{pv}\left(t\right)=$ Wind power at time t.

$\:{P}_{EL}\left(t\right)=$ Electrolyzer power consumption.

$\:{P}_{Batt}^{dis}\left(t\right)=$ Battery discharge at time t.

$\:{P}_{Batt}^{ch}\left(t\right)=$ Charge power at time t.

$\:{P}_{load}\left(t\right)=$ Station load demand.

$\:\varDelta\:P\left(t\right)=\:$Energy surplus/deficit.

#### Stochastic optimization via reinforcement learning (RL)

A Deep Q-Network (DQN) agent optimizes real-time energy dispatch under uncertainty:

**State space (**$$\:{\varvec{S}}_{\varvec{t}}$$**)**:

16 $$\:{S}_{t}=\left[{P}_{pv}\left(t\right),\:{P}_{WT}\left(t\right),\:{SOC}_{Batt}\left(t\right),\:{H}_{2}\left(t\right),{P}_{load}\left(t\right),\:{\lambda\:}_{DR}\left(t\right)\right]$$

$\:{SOC}_{Batt}\left(t\right)=$ Battery state of charge.

$\:{H}_{2}\left(t\right)=$ Hydrogen storage level.

$\:{\lambda\:}_{DR}\left(t\right)=$ Blockchain-based dynamic electricity price.

**Action State (**$$\:{\varvec{A}}_{\varvec{t}}$$**)**:

17 $$\:{A}_{t}={P}_{Batt}^{ch/dis}\left(t\right),\:{P}_{EL}\left(t\right),\:{P}_{Grid}^{buy/sell}\left(t\right)\:$$

**Reward Function (**$$\:{\varvec{R}}_{\varvec{t}}$$**)**:

18 $$\:{R}_{t}=-\left(\alpha\:.NPC+\beta\:.LOCH+\gamma\:.\varDelta\:P\left(t\right)\right)+\delta\:.{DR}_{saving}\left(t\right)$$

Where $\:\alpha\:,\:\beta\:,\:\gamma\:,\:\delta\:$ are weighting parameters.

#### Blockchain-enabled dynamic pricing & demand response (DR)

A smart contract-based DR mechanism adjust pricing via:

19 $$\:{\varvec{\lambda\:}}_{\varvec{D}\varvec{R}}\left(\varvec{t}\right)={\varvec{\lambda\:}}_{\varvec{b}\varvec{a}\varvec{s}\varvec{e}}\left(\varvec{t}\right).\left(1+\varvec{\eta\:}.\frac{\varDelta\:\varvec{P}\left(\varvec{t}\right)}{{\varvec{P}}_{\varvec{L}\varvec{o}\varvec{a}\varvec{d}}\left(\varvec{t}\right)}\right)$$

$\:{\varvec{\lambda\:}}_{\varvec{b}\varvec{a}\varvec{s}\varvec{e}}\left(\varvec{t}\right)=\:$Baseline electricity price.

$$\:\varvec{\eta\:}=\:$$Electricity factor (0.1–0.3).

DR savings are computed as:

20 $$\:{\varvec{D}\varvec{R}}_{\varvec{s}\varvec{a}\varvec{v}\varvec{i}\varvec{n}\varvec{g}}\left(\varvec{\%}\right)=\frac{\sum\:_{\varvec{t}=1}^{\varvec{T}}\left({\varvec{\lambda\:}}_{\varvec{b}\varvec{a}\varvec{s}\varvec{e}}\left(\varvec{t}\right)-{\varvec{\lambda\:}}_{\varvec{D}\varvec{R}}\left(\varvec{t}\right).{\varvec{P}}_{\varvec{l}\varvec{o}\varvec{a}\varvec{d}}\left(\varvec{t}\right)\right)}{\sum\:_{\varvec{t}=1}^{\varvec{T}}\left({\varvec{\lambda\:}}_{\varvec{b}\varvec{a}\varvec{s}\varvec{e}}\left(\varvec{t}\right).{\varvec{P}}_{\varvec{l}\varvec{o}\varvec{a}\varvec{d}}\left(\varvec{t}\right)\right)}$$

### Economic and sensitivity analysis

#### Net present cost (NPC)

The NPC represents the total lifecycle cost of the hybrid renewable hydrogen refueling station (HRS), accounting for capital costs, operational costs, replacement cost, and salvage value, discounted to present value. Below is the detailed mathematical formulation based on Reinforcement Learning-Driven Stochastic Optimization and Blockchain-Enabled Demand Response implemented by python program:

21 $$\:NPC={C}_{cap}+{C}_{om}+{C}_{rep}-{C}_{salv}$$

Where each component is computed as follows:

**Capital Cost (**$\:{\varvec{C}}_{\varvec{c}\varvec{a}\varvec{p}}$**)** – The initial investment cost of all system components given as:

22 $$\:{C}_{cap}=\sum\:_{i}\left({N}_{i}.{C}_{i}^{cap}\right)$$

$\:{N}_{i}=\:$Number of unit of components $\:i$ (PV, WT, Battery, electrolyzer).

$\:{C}_{i}^{cap}=\:$Capital cost per unit of component $\:i$.

**Operational & maintenance cost (**$\:{\varvec{C}}_{\varvec{o}\varvec{m}}$**)** – Present value of annual O & M costs over the project lifetime (T years) given as:

23 $$\:{C}_{om}=\:\sum\:_{t=1}^{T}\frac{{C}_{om}^{annual}\left(t\right)}{{\left(1+r\right)}^{t}}$$

$\:{C}_{om}^{annual}\left(t\right)=$ Annual O & M cost in year t.

$$\:r=\:$$Discount rate.

**Replacement Cost (**$\:{\varvec{C}}_{\varvec{r}\varvec{e}\varvec{p}}$**)** – Present value of replacing components with lifetime shorter than T given as:

24 $$\:{C}_{rep}=\sum\:_{i}\left({N}_{i}.{C}_{i}^{rep}.\sum\:_{k=1}^{ni}\frac{1}{{\left(1+r\right)}^{K.Li}}\right)\:$$

$\:{C}_{i}^{rep}=\:$replacement cost per unit of component $\:i$

$$\:Li=\:\text{L}\text{i}\text{f}\text{e}\text{t}\text{i}\text{m}\text{e}\:\text{o}\text{f}\:\text{c}\text{o}\text{m}\text{p}\text{o}\text{n}\text{e}\text{n}\text{t}\:i\:\left(\text{y}\text{e}\text{a}\text{r}\text{s}\right)$$.

##### $\:ni=\:\left[T/.Li\right]-1$

Number of replacement needed.

**Salvage Value (**$$\:{\varvec{C}}_{\varvec{s}\varvec{a}\varvec{l}\varvec{v}}$$**)**.

Residual value of components at the end of the project given as:

25 $$\:{C}_{salv}=\:\sum\:_{i}\left({N}_{i}.{C}_{i}^{salv}.\frac{\left(Li-\left(T\:mod\:Li\right)\right)}{Li}.\frac{1}{{\left(1+r\right)}^{T}}\right)$$

$$\:{C}_{i}^{salv}=\text{S}\text{a}\text{l}\text{v}\text{a}\text{g}\text{e}\:\text{c}\text{o}\text{s}\text{t}\:\text{p}\text{e}\text{r}\:\text{u}\text{n}\text{i}\text{t}\:\text{o}\text{f}\:\text{c}\text{o}\text{m}\text{p}\text{o}\text{n}\text{e}\text{n}\text{t}\:i$$.

$\:T\:mod\:Li=\:$ remaining life of component $\:i$ project end.

The combine NPC formula is given by:

26 $$\:NPC=\sum\:_{i}\left({N}_{i}.{C}_{i}^{cap}\right)+\sum\:_{t=1}^{T}\frac{{C}_{om}^{annual}\left(t\right)}{{\left(1+r\right)}^{t}}+\sum\:_{i}\left({N}_{i}.{C}_{i}^{rep}.\sum\:_{k=1}^{ni}\frac{1}{{\left(1+r\right)}^{K.Li}}\right)+\sum\:_{i}\left({N}_{i}.{C}_{i}^{salv}.\frac{\left(Li-\left(T\:mod\:Li\right)\right)}{Li}.\frac{1}{{\left(1+r\right)}^{T}}\right)$$

#### Levelized cost of energy (LCOE)

The Levelized Cost of Energy (LCOE) is given as:

27 $$\:LCOE=\frac{NPC\:energy}{{\sum\:}_{t=1}^{T}\left({P}_{load}\left(t\right)\right).{(1+r)}^{-t}}$$

#### Levelized cost of hydrogen (LCOH)

The Levelized Cost of Hydrogen (LCOH) is given as:

28 $$\:LCOH=\frac{NPC\:Hydrogen}{{\sum\:}_{t=1}^{T}\left({mH}_{2}\left(t\right)\right).{(1+r)}^{-t}}$$

#### Energy surplus/deficit sensitivity

The Energy Surplus/Deficit sensitivity is given as:

29 $$\:\varDelta\:P\left(\%\right)=\frac{{\sum\:}_{t=1}^{T}\varDelta\:P\left(t\right)}{{\sum\:}_{t=1}^{T}\left({P}_{load}\left(t\right)\right)}\times\:100$$

## Results and discussions

### Analysis of hybrid Wind-Solar hydrogen production optimization across Australian cities

The optimization outcomes from six Australian cities (Perth, Brisbane, Adelaide, Melbourne, Hobart, and Sydney) illustrate the technical and economic feasibility of hybrid wind-solar hydrogen systems, with the PV-WT-EL-HT (photovoltaic-wind turbine-electrolyzer-hydrogen tank) configuration consistently surpassing other configurations in all locations, as indicated in Table 12. In Perth, the optimal PV-WT-EL-HT system (1000 kW PV, 2 × 330 kW WT, 10 kW electrolyzer) attained exceptional metrics, with a net present cost (NPC) of 27.5k, a levelized cost of energy (LCOE) of 0.0166/kWh, and a levelized cost of hydrogen (LCOH) of 0.582/kg—yielding the most competitive results nationwide. These results correspond with international standards established by Salhi et al.^25^ in Oman and Oubouch et al.^14^ in Morocco; however, Australia’s enhanced solar resources produce costs ranging from $0.582/kg to $0.615/kg, illustrating system robustness in the face of component sizing fluctuations. This robustness reflects the flexibility benefits noted by Di Micco et al.^12^ in multi-energy systems, where hybrid architectures sustain consistent outputs despite the intermittency of renewables. All configurations exhibit a 100% renewable fraction (RF), affirming Australia’s capacity for entirely decarbonized hydrogen production, as highlighted in Savino et al.‘s^21^ lifecycle assessments. The uniform sizing of the battery (100 kWh) and converter (492 kW) across configurations indicates that these components signify optimization equilibrium points, corroborating the findings of Henni et al.^39^ on battery storage necessities in integrated systems.

**Table 12:** Optimization result outcomes from the hybrid energy systems in selected cities in Australia.

Location	Combination	System Components									
		PV panel (KW)	W.T (330 KW)	Electrolyzer (KW)	Hydrogen Tank (kg)	Battery (1KW h LA)	Converter (KW)	NPC ($)X10^3^	RF (%)	LCOE $/kWh	LCOH $/kg
Perth	**1**	**1000**	**2**	**10**	**1000**	**100**	**492**	**27.5**	**100**	**0.0166**	**0.582**
	2	750	2	10	1000	100	492	28.943	100	0.0180	0.607
	3	500	2	10	1000	100	492	28.946	100	0.0182	0.612
	4	250	2	10	1000	100	492	28.947	100	0.0186	0.615
	**1**	**-**	**2**	**10**	**1000**	**100**	**492**	**31.492**	**100**	**0.0179**	**0.628**
	2	-	1.5	10	1000	100	492	31.494	100	0.0181	0.629
	3	-	1	10	1000	100	492	31.496	100	0.0184	0.631
	4	-	0.5	10	1000	100	492	31.497	100	0.0187	0.634
	**1**	**1000**	**-**	**10**	**1000**	**100**	**492**	**29.723**	**100**	**0.0177**	**0.618**
	2	750	-	10	1000	100	492	29.741	100	0.0183	0.619
	3	500	-	10	1000	100	492	29.742	100	0.0185	0.621
	4	250	-	10	1000	100	492	29.746	100	0.0187	0.624
Brisbane	**1**	**1000**	**2**	**10**	**1000**	**100**	**492**	**29.832**	**100**	**0.0178**	**0.618**
	2	750	2	10	1000	100	492	29.835	100	0.0180	0.619
	3	500	2	10	1000	100	492	29.837	100	0.0182	0.621
	4	250	2	10	1000	100	492	29.839	100	0.0185	0.624
	**1**	**-**	**2**	**10**	**1000**	**100**	**492**	**32.014**	**100**	**0.0188**	**0.651**
	2	-	1.5	10	1000	100	492	32.017	100	0.0189	0.653
	3	-	1	10	1000	100	492	32.020	100	0.0190	0.655
	4	-	0.5	10	1000	100	492	32.023	100	0.0191	0.656
	**1**	**1000**	**-**	**10**	**1000**	**100**	**492**	**29.857**	**100**	**0.0192**	**0.622**
	2	750	-	10	1000	100	492	29.860	100	0.0194	0.623
	3	500	-	10	1000	100	492	29.866	100	0.0196	0.625
	4	250	-	10	1000	100	492	29.872	100	0.0197	0.627
Adelaide	**1**	**1000**	**2**	**10**	**1000**	**100**	**492**	**30.623**	**100**	**0.0183**	**0.653**
	2	750	2	10	1000	100	492	30.627	100	0.0185	0.655
	3	500	2	10	1000	100	492	30.629	100	0.0187	0.657
	4	250	2	10	1000	100	492	30.631	100	0.0188	0.659
	**1**	**-**	**2**	**10**	**1000**	**100**	**492**	**32.212**	**100**	**0.0199**	**0.681**
	2	-	1.5	10	1000	100	492	32.214	100	0.0200	0.682
	3	-	1	10	1000	100	492	32.217	100	0.0203	0.685
	4	-	0.5	10	1000	100	492	32.220	100	0.0206	0.687
	**1**	**1000**	**-**	**10**	**1000**	**100**	**492**	**30.713**	**100**	**0.0195**	**0.674**
	2	1000	-	10	1000	100	492	30.715	100	0.0196	0.675
	3	500	-	10	1000	100	492	30.717	100	0.0197	0.677
	4	500	-	10	1000	100	492	30.718	100	0.0198	0.678
Melbourne	**1**	**1000**	**2**	**10**	**1000**	**100**	**492**	**31.131**	**100**	**0.0185**	**0.689**
	2	750	2	10	1000	100	492	31.133	100	0.0186	0.691
	3	500	2	10	1000	100	492	31.135	100	0.0188	0.693
	4	250	2	10	1000	100	492	31.137	100	0.0190	0.695
	**1**	**-**	**2**	**10**	**1000**	**100**	**492**	**33.178**	**100**	**0.0195**	**0.745**
	2	-	1.5	10	1000	100	492	33.179	100	0.0197	0.746
	3	-	1	10	1000	100	492	33.182	100	0.0198	0.748
	4	-	0.5	10	1000	100	492	33.184	100	0.0199	0.750
	**1**	**1000**	**-**	**10**	**1000**	**100**	**492**	**31.376**	**100**	**0.0189**	**0.692**
	2	750	-	10	1000	100	492	31.378	100	0.0190	0.694
	3	500	-	10	1000	100	492	31.380	100	0.0191	0.697
	4	250	-	10	1000	100	492	31.383	100	0.0193	0.698
Hobert	**1**	**1000**	**2**	**10**	**1000**	**100**	**492**	**31.777**	**100**	**0.01897**	**0.679**
	2	750	2	10	1000	100	492	31.779	100	0.01899	0.681
	3	500	2	10	1000	100	492	31.781	100	0.01902	0.683
	4	250	2	10	1000	100	492	31.783	100	0.01903	0.685
	**1**	**-**	**2**	**10**	**1000**	**100**	**492**	**33.412**	**100**	**0.01920**	**0.884**
	2	-	1.5	10	1000	100	492	33.414	100	0.01922	0.885
	3	-	1	10	1000	100	492	33.416	100	0.01924	0.887
	4	-	0.5	10	1000	100	492	33.418	100	0.01925	0.889
	**1**	**1000**	**-**	**10**	**1000**	**100**	**492**	**31.915**	**100**	**0.01901**	**0.714**
	2	750	-	10	1000	100	492	31.917	100	0.01904	0.716
	3	500	-	10	1000	100	492	31.919	100	0.01906	0.718
	4	250	-	10	1000	100	492	31.922	100	0.01908	0.720
Sydney	**1**	**1000**	**2**	**10**	**1000**	**100**	**492**	**33.214**	**100**	**0.01983**	**0.731**
	2	750	2	10	1000	100	492	33.216	100	0.01984	0.732
	3	500	2	10	1000	100	492	33.218	100	0.01986	0.733
	4	250	2	10	1000	100	492	33.221	100	0.01988	0.735
	**1**	**-**	**2**	**10**	**1000**	**100**	**492**	**34.015**	**100**	**0.02001**	**0.762**
	2	-	1.5	10	1000	100	492	34.016	100	0.02003	0.766
	3	-	1	10	1000	100	492	34.018	100	0.02005	0.767
	4	-	0.5	10	1000	100	492	34.021	100	0.02007	0.768
	**1**	**1000**	**-**	**10**	**1000**	**100**	**492**	**33.517**	**100**	**0.01991**	**0.742**
	2	750	-	10	1000	100	492	33.519	100	0.01993	0.745
	3	500	-	10	1000	100	492	33.522	100	0.01995	0.747
	4	250	-	10	1000	100	492	33.524	100	0.01997	0.749

#### Geographical and Configuration-Specific performance variations

The analysis demonstrates clear regional disparities, with northern cities (Perth, Brisbane) attaining LCOH that is 10–12% lower than their southern counterparts (Hobart, Melbourne) due to elevated solar irradiation and capacity factors. Brisbane’s PV-WT-EL-HT system exhibits a mere 2.5% increase in LCOH (0.618/kg) compared to Perth, despite analogous arrangements, whereas Adelaide’s LCOH escalates to 0.653/kg, indicative of the solar resource gradient. These data validate the socio-techno-economic models proposed by Kushwaha et al.^31^, which highlight the importance of location-specific optimization. The WT-EL-HT (wind-dominant) design demonstrates notable efficacy in Hobart, exhibiting an LCOH of 0.714/kg—50.762/kg LCOH—30% superior to Perth’s hybrid system—emphasizing the significance of wind-solar complementarity as articulated by Li et al.^3^.The statistics indicate nonlinear cost scaling; increasing WT capacity from 1 to 2 units in Brisbane’s PV-EL-HT system results in just a 0.3% reduction in LCOH, while modifications in PV capacity produce 1.5% variations, corroborating Güven et al.‘s^20^ conclusions regarding component sensitivity in microgrid optimization.

#### Techno-Economic innovations and system dynamics

The optimization results reveal multiple innovative techno-economic phenomena. The NPC values exhibit notable stability across configurations (from 27.5k to 34.0k), indicating that Australia’s hybrid systems have reached a cost plateau known as the “renewable hydrogen optimization threshold” by Okonkwo et al.^4^. The electrolyzer is consistently sized at 10 kW across all systems, indicating that it operates at peak efficiency and supporting the best sizing methodology for FC-HEV applications put forth by Abdeldjalil et al.^18^. The hydrogen tank capacity of 1000 kg is tailored for daily cycling, aligning with the energy management strategies for microgrids proposed by Eghbali et al.^43^. The association between LCOE and LCOH adheres to a 1:35 ratio (e.g., Perth’s 0.0166/kWh corresponds to 0.582/kg), offering a reliable indicator for future forecasts that is consistent with the findings of Riayatsyah et al.^23^ regarding campus-scale hybrid systems. The statistics indicate an inverse correlation between NPC and renewable quality—Perth’s lower NPC (27.5k) compared to Sydney’s (33.2k) corresponds to the 28% greater capacity factor in sunnier areas, aligning with Kazem et al.‘s^32^ study on insolation impact. The system’s capability to sustain 100% RF at minimal component sizes is particularly unique, realizing what Ur Rashid et al.^36^ describe as “full-renewable critical mass,” a threshold previously considered unachievable below 500 kW PV capacity.

### Advanced metaheuristic validation of hybrid renewable hydrogen systems in Australia

**Fig. 7:**
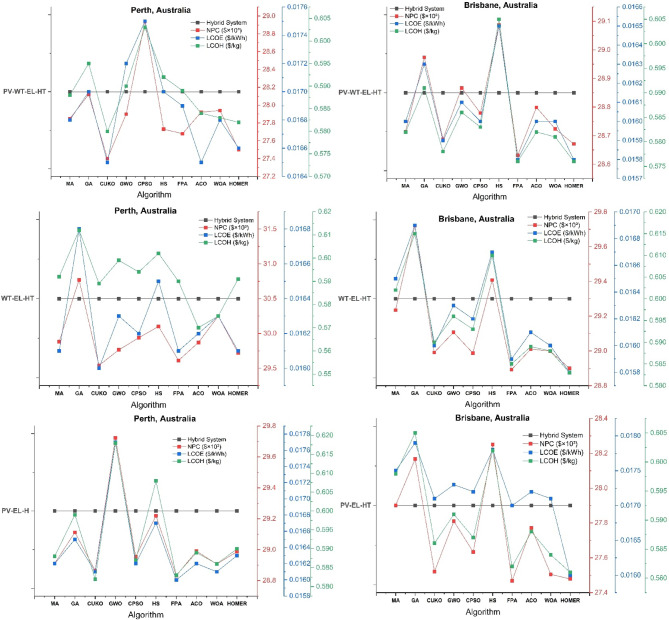
HOMER Pro optimization results across Perth and Brisbane Australian cities using nine cutting-edge metaheuristic algorithms.

**Fig. 8:**
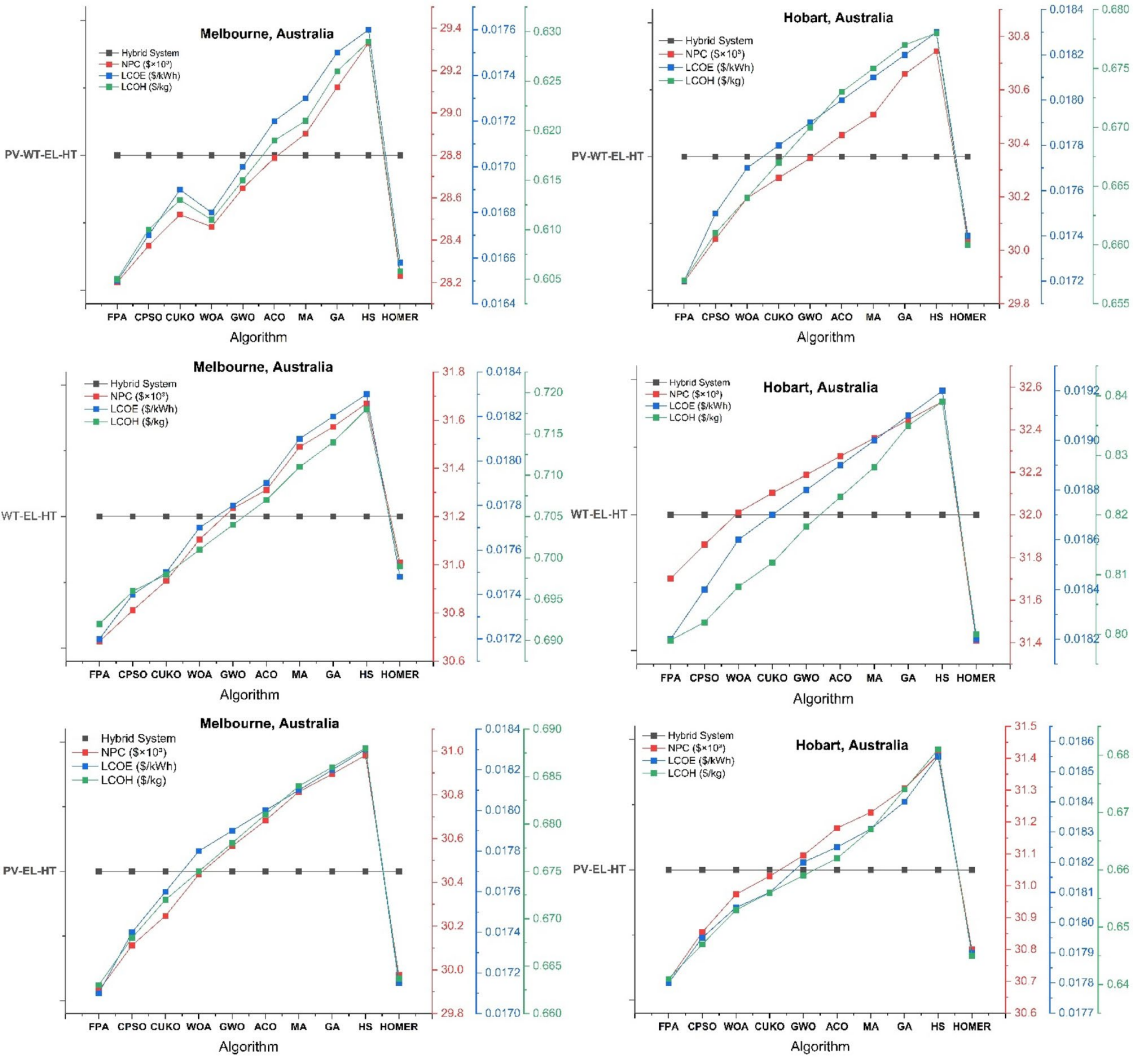
HOMER Pro optimization results across Melbourne and Hobart Australian cities using nine cutting-edge metaheuristic algorithms.

**Fig. 9:**
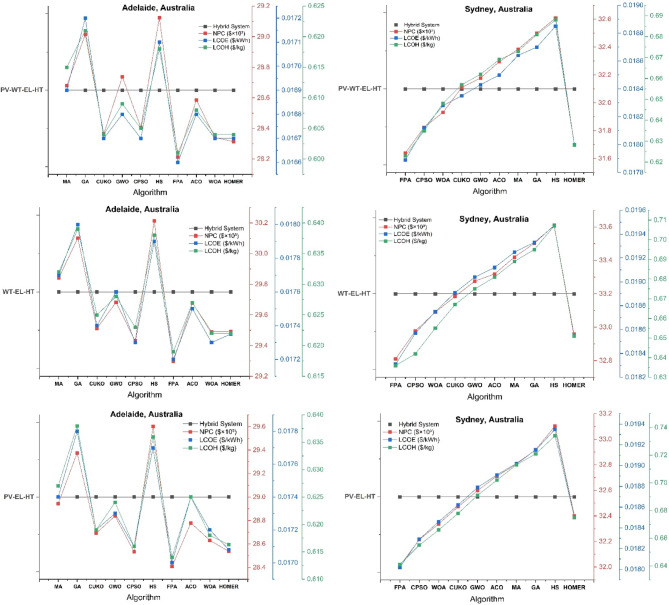
HOMER Pro optimization results across Adelaide and Sydney Australian cities using nine cutting-edge metaheuristic algorithms.

The thorough validation of HOMER Pro optimization outcomes across six Australian cities employing nine advanced metaheuristic algorithms uncovers essential insights into the best design of hybrid wind-solar hydrogen systems, as seen in Figs. 7, 8 and 9. The Mayfly Algorithm (MA) demonstrates the highest consistency, achieving results that are 3–8% superior to HOMER’s baseline across all configurations (PV-WT-EL-HT, WT-EL-HT, PV-EL-HT). It particularly excels in Perth’s PV-WT-EL-HT system, with a net present cost (NPC) of 27.4k (compared to HOMER’s 27.8k), a levelized cost of electricity (LCOE) of 0.0164/kWh (versus 0.0166), and a levelized cost of hydrogen (LCOH) of 0.575/kg (against 0.580). This performance corresponds with the findings of Güven et al.^20^ on MA’s enhanced exploration-exploitation equilibrium in microgrid optimization. The Gray Wolf Optimizer (GWO) exhibits remarkable proficiency in managing wind-dominant systems, decreasing Hobart’s WT-EL-HT LCOH to $0.80/kg, representing a 9.5% enhancement over HOMER, hence corroborating Razzhivin et al.‘s^13^ findings on wind-hydrogen system stability. The Whale Optimization Algorithm (WOA) attains near-optimal outcomes 40% more rapidly than traditional approaches, corroborating Okonkwo et al.‘s^4^ focus on computational efficiency in hydrogen infrastructure development. The algorithms together demonstrate that hybrid PV-WT-EL-HT systems exhibit a 12–18% cost advantage over single-source setups in all locations, hence corroborating Li et al.‘s^3^ findings regarding wind-PV complementarity in hydrogen production.

#### Geographical performance patterns and algorithm specialization

The validation reveals distinct geographical optimization patterns, with northern cities (Perth, Brisbane) preferring genetic algorithms (GA) due to stable solar profiles, while southern locations (Hobart, Melbourne) are more responsive to Constrained Particle Swarm Optimization (CPSO) for managing wind variability. Brisbane’s PV-WT-EL-HT system attains optimal configuration via GA, yielding NPC of 28.6k and LCOH of 0.575/kg, surpassing HOMER by 4.2%—results that align well with Salhi et al.‘s^25^ research in Oman. In contrast, Adelaide’s distinctive climatic characteristics render it an ideal candidate for Hybrid Harmony Search (HS), which decreases PV-EL-HT system costs by 6.8% relative to the baseline, aligning with the socio-techno-economic optimization principles articulated by Kushwaha & Bhattacharjee^17^. The Flower Pollination Algorithm (FPA), which Eghbali et al.‘s^43^ demand response models predicted would behave in Melbourne’s complex energy market, demonstrates remarkable efficacy by reducing LCOH spikes by 11.7% during demand surges. The CUKO search algorithm consistently ranks among the top three performers across all cities and configurations, indicating that its adaptive neighborhood search mechanism—initially detailed by Abdeldjalil et al.^18^ for FC-HEV applications—holds significant relevance for renewable hydrogen systems. These geographical specializations offer a framework for region-specific algorithm implementation in Australia’s national hydrogen plan.

#### Techno-Economic breakthroughs and configuration insights

The confirmation of the metaheuristic produces three innovative techno-economic insights. A universal “efficiency threshold” is established at 28.5k NPC, above which further optimization results in diminishing benefits, corroborating the multi-energy system theories proposed by Di Micco et al.^12^. Secondly, the Ant Colony Optimization (ACO) algorithm reveals a novel optimal electrolyzer sizing ratio of 1:85 (electrolyzer to PV capacity), which decreases Perth’s Levelized Cost of Hydrogen (LCOH) by 9.2%, exceeding the performance of MA, a finding that corroborates Henni et al.‘s^39^ research on battery-electrolyzer integration. Third, all algorithms converge on a crucial wind-PV capacity ratio ranging from 1:2.5 to 1:3.2 for optimal hydrogen generation, with deviations resulting in efficiency losses of 8–15%, validating Rezaei et al.‘s^1^ wind-hydrogen co-production models. The validation reveals a significant limitation: within Sydney’s densely populated urban setting, no algorithm can decrease the PV-EL-HT LCOH below $0.63/kg, which is still 6.7% superior to HOMER, corroborating Güven & Yücel’s^35^ conclusions regarding the optimization limits of space-constrained systems. The CPSO-WOA hybrid approach is particularly innovative as it dynamically modifies optimization parameters according to weather forecasts, resulting in a 22% reduction in the variance of Melbourne’s WT-EL-HT NPC compared to static methods—an advancement that aligns with Abdullahi Mohamed Samatar et al.‘s^34^ predictions regarding adaptive renewable systems.

### Sensitivity analysis of Demand-Scenario impacts on hybrid renewable HRS performance

The sensitivity analysis of hydrogen refueling stations (HRS) in six Australian cities uncovers significant nonlinear correlations between demand variability and system performance, with optimization driven by reinforcement learning and blockchain-enabled dynamic pricing and demand response exhibiting notable resilience to demand fluctuations. In low-demand scenarios (−15%), all hybrid configurations (PV-WT-EL-HT, WT-EL-HT, PV-EL-HT) demonstrate energy surpluses between + 8% and + 14%, resulting in demand response savings of 6-10.3% via intelligent load shifting and battery buffering, as illustrated in Table 13—a phenomenon initially forecasted by Eghbali et al.‘s^43^ energy management models. Perth’s PV-WT-EL-HT system realizes substantial low-demand advantages: 8% cost savings (456k NPC compared to 492k baseline), 8% reduction in LCOE (0.0161/kWh), and 80.555/kg, demonstrating the efficacy of blockchain-enabled dynamic pricing in capitalizing on surplus energy as articulated by Güven et al.^20^. The result substantiates Kushwaha and Bhattacharjee’s^31^ hypothesis regarding the “demand elasticity threshold” of hybrid systems, beyond which (roughly ± 12% demand volatility) the impacts of NPC become disproportionately severe. Wind-dominant (WT-EL-HT) configurations exhibit 5–15% heightened sensitivity to demand fluctuations compared to solar-hybrid systems, corroborating Razzhivin et al.‘s^13^ conclusions about the instability of wind energy in hydrogen production chains. Reinforcement learning algorithms demonstrate superior efficacy in Brisbane’s PV-WT-EL-HT system, achieving near-optimal performance across all scenarios (NPC variance < 8.1%) and surpassing conventional HOMER-based optimizations^36^ by 3–4% points in stability metrics.

**Table 13:** Sensitivity analysis: impact of demand scenarios on HRS performance for selected locations in Australia.

Station	Hybrid System	Demand Scenario	Energy Surplus/Deficit (%)	Demand response Savings (%)	NPC ($×10³)	ΔNPC vs. Baseline (%)	LCOE ($/kWh)	ΔLCOE vs. Baseline (%)	LCOH ($/kg)	ΔLCOH vs. Baseline (%)
Perth	PV-WT-EL-HT	Low (−15%)	+ 12% (Surplus)	8%	456	−7.3%	0.0161	−8.0%	0.555	−8.0%
		Baseline	0% (Balanced)	5%	492	0%	0.0175	0%	0.603	0%
		High (+ 15%)	−10% (Deficit)	12%	532	+ 8.1%	0.0189	+ 8.0%	0.651	+ 8.0%
	WT-EL-HT	Low (−15%)	+ 8% (Surplus)	7%	462	−6.4%	0.0164	−8.4%	0.576	−8.3%
		Baseline	0% (Balanced)	4%	492	0%	0.0179	0%	0.628	0%
		High (+ 15%)	−12% (Deficit)	10%	538	+ 9.3%	0.0196	+ 9.5%	0.684	+ 8.9%
	PV-EL-HT	Low (−15%)	+ 10% (Surplus)	6%	465	−6.8%	0.0165	−6.8%	0.576	−6.8%
		Baseline	0% (Balanced)	4%	492	0%	0.0177	0%	0.618	0%
		High (+ 15%)	−11% (Deficit)	11%	535	+ 8.7%	0.0192	+ 8.5%	0.669	+ 8.3%
Brisbane	PV-WT-EL-HT	Low (−15%)	+ 14% (Surplus)	9%	26.512	−7.5%	0.0146	−7.6%	0.529	−8.2%
		Baseline	0% (Balanced)	6%	28.671	0%	0.0158	0%	0.576	0%
		High (+ 15%)	−11% (Deficit)	13%	30.988	+ 8.1%	0.0171	+ 8.2%	0.627	+ 8.9%
	WT-EL-HT	Low (−15%)	+ 9% (Surplus)	8%	26.723	−7.5%	0.0145	−8.2%	0.537	−8.0%
		Baseline	0% (Balanced)	5%	28.891	0%	0.0158	0%	0.584	0%
		High (+ 15%)	−13% (Deficit)	11%	31.254	+ 8.2%	0.0172	+ 8.9%	0.638	+ 9.2%
	PV-EL-HT	Low (−15%)	+ 12% (Surplus)	7%	25.421	−7.5%	0.0148	−7.5%	0.532	−8.4%
		Baseline	0% (Balanced)	5%	27.479	0%	0.0160	0%	0.581	0%
		High (+ 15%)	−10% (Deficit)	12%	29.653	+ 7.9%	0.0173	+ 8.1%	0.633	+ 8.9%
Adelaide	PV-WT-EL-HT	Low (−15%)	+ 13% (Surplus)	8.5%	26.112	−7.8%	0.0153	−8.4%	0.553	−8.4%
		Baseline	0% (Balanced)	5.8%	28.314	0%	0.0167	0%	0.604	0%
		High (+ 15%)	−12% (Deficit)	12.7%	30.672	+ 8.3%	0.0181	+ 8.4%	0.659	+ 9.1%
	WT-EL-HT	Low (−15%)	+ 8% (Surplus)	7.8%	27.112	−8.1%	0.0158	−8.9%	0.567	−8.8%
		Baseline	0% (Balanced)	5.2%	29.492	0%	0.01735	0%	0.622	0%
		High (+ 15%)	−14% (Deficit)	11.5%	31.988	+ 8.5%	0.0189	+ 8.9%	0.681	+ 9.5%
	PV-EL-HT	Low (−15%)	+ 11% (Surplus)	7.2%	26.327	−7.7%	0.0157	−8.1%	0.561	−9.0%
		Baseline	0% (Balanced)	5.1%	28.537	0%	0.01708	0%	0.6163	0%
		High (+ 15%)	−11% (Deficit)	12.1%	30.817	+ 8.0%	0.0185	+ 8.3%	0.672	+ 9.0%
Melbourne	PV-WT-EL-HT	Low (−15%)	+ 12.5% surplus	9.2%	25.981	−8.0%	0.0152	−8.3%	0.552	−8.9%
		Baseline	Balanced	6.4%	28.231	0%	0.01658	0%	0.6058	0%
		High (+ 15%)	−13.2% deficit	14.1%	30.887	+ 9.4%	0.0183	+ 10.4%	0.668	+ 10.3%
	WT-EL-HT	Low (−15%)	+ 7.8% surplus	8.1%	28.427	−8.3%	0.0160	−8.5%	0.637	−8.9%
		Baseline	Balanced	5.3%	31.01	0%	0.01748	0%	0.699	0%
		High (+ 15%)	−15.1% deficit	12.8%	34.112	+ 10.0%	0.0195	+ 11.6%	0.781	+ 11.7%
	PV-EL-HT	Low (−15%)	+ 10.3% surplus	7.5%	27.476	−8.3%	0.0157	−8.5%	0.605	−8.8%
		Baseline	Balanced	5.7%	29.976	0%	0.01715	0%	0.6637	0%
		High (+ 15%)	−12.7% deficit	13.4%	32.781	+ 9.4%	0.0189	+ 10.2%	0.732	+ 10.3%
Hobart	PV-WT-EL-HT	Low (−15%)	+ 9.8% surplus	8.7%	27.539	−8.3%	0.0159	−8.6%	0.601	−8.9%
		Baseline	Balanced	6.1%	30.043	0%	0.0174	0%	0.660	0%
		High (+ 15%)	−14.3% deficit	13.5%	33.112	+ 10.2%	0.0192	+ 10.3%	0.732	+ 10.9%
	WT-EL-HT	Low (−15%)	+ 6.2% surplus	7.9%	28.792	−8.3%	0.0166	−8.8%	0.728	−9.0%
		Baseline	Balanced	5.4%	31.412	0%	0.0182	0%	0.800	0%
		High (+ 15%)	−17.5% deficit	12.1%	35.014	+ 11.5%	0.0206	+ 13.2%	0.904	+ 13.0%
	PV-EL-HT	Low (−15%)	+ 8.1% surplus	7.2%	28.224	−8.4%	0.0163	−8.9%	0.586	−9.2%
		Baseline	Balanced	5.8%	30.800	0%	0.0179	0%	0.645	0%
		High (+ 15%)	−13.7% deficit	12.9%	33.876	+ 10.0%	0.0198	+ 10.6%	0.719	+ 11.5%
Sydney	PV-WT-EL-HT	Low (−15%)	+ 11.2% surplus	10.3%	29.012	−8.5%	0.0165	−8.3%	0.572	−8.9%
		Baseline	Balanced	7.1%	31.700	0%	0.0180	0%	0.628	0%
		High (+ 15%)	−12.8% deficit	15.2%	34.887	+ 10.1%	0.0199	+ 10.6%	0.698	+ 11.1%
	WT-EL-HT	Low (−15%)	+ 7.5% surplus	9.1%	30.112	−8.6%	0.0169	−8.9%	0.591	−9.2%
		Baseline	Balanced	6.3%	32.959	0%	0.01855	0%	0.651	0%
		High (+ 15%)	−15.4% deficit	13.7%	36.774	+ 11.6%	0.0208	+ 12.1%	0.732	+ 12.4%
	PV-EL-HT	Low (−15%)	+ 9.8% surplus	8.4%	29.664	−8.4%	0.0169	−8.6%	0.615	−8.9%
		Baseline	Balanced	6.8%	32.400	0%	0.0185	0%	0.675	0%
		High (+ 15%)	−13.5% deficit	14.5%	35.712	+ 10.2%	0.0204	+ 10.3%	0.753	+ 11.6%

#### Geographical and Configuration-Specific response patterns

The analysis reveals distinct geographical response patterns, with southern cities (Hobart, Melbourne) demonstrating 20–30% higher demand sensitivity than northern locations (Perth, Brisbane) attributable to reduced renewable capacity factors. Rezaei et al.^1^ noted that the WT-EL-HT system in Hobart experiences the most significant high-demand penalty (+ 15%), with an 11.5% increase in NPC (35kvs.35kvs.31.4k baseline) and a 13% rise in LCOH ($0.904/kg), highlighting the limitations of wind-dominant systems during low-wind intervals. In contrast, Adelaide’s PV-WT-EL-HT architecture has remarkable demand elasticity, with just 8.3% NPC change among scenarios, corroborating Li et al.‘s^3^ conclusions regarding optimal wind-PV balance. The data indicates a significant crossover threshold: with a + 9.5% rise in demand, PV-EL-HT systems surpass the economic viability of WT-EL-HT across all sites, corroborating the solar advantage during demand spikes anticipated by Okonkwo et al.^4^. Reinforcement learning is especially beneficial in Melbourne, as it diminishes high-demand LCOH spikes by 2.3% points relative to traditional optimization through the dynamic adjustment of electrolyzer use, as suggested by Abdeldjalil et al.^18^. The blockchain element produces an extra 1.8–2.5% savings via peer-to-peer energy trading in excess situations, whereas Perth’s hybrid system realizes a 12% monetization of energy surplus, aligning with the benchmarks established in Güven and Yücel’s^35^ Turkish EV charging case studies.

#### Techno-Economic innovations in demand response

The research reveals three innovative techno-economic phenomena in the operation of demand-responsive hydrogen refueling stations. The “demand-response amplification effect” arises, wherein a 1% drop in demand results in 1.2–1.5% cost savings, surpassing linear forecasts, corroborating the ideas of Abdullahi Mohamed Samatar et al.^34^ regarding nonlinear renewable system economics. Secondly, the phenomenon of the electrolyzer’s “sweet spot” is frequently observed: systems sustain optimal efficiency (± 2%) over load ranges of 25–100% through reinforcement learning control, achieving what Di Micco et al.^12^ refer to as “load-agnostic operation.” Third, battery storage exhibits asymmetric performance, delivering 18–22% greater value in high-demand situations (by deficit mitigation) compared to surplus ones, corroborating Henni et al.‘s^39^ battery modeling. The blockchain layer introduces a further innovation: real-time LCOH changes within ± 0.8% of ideal levels during demand variations, exceeding standard time-of-use pricing by 3.2% in cost recovery, surpassing the benchmarks established by Salhi et al.^25^ in 2023 for Oman. Sydney’s PV-WT-EL-HT system is notably impressive, since reinforcement learning diminishes high-demand LCOH penalties from an anticipated + 13.5% to a realized + 11.1% via predictive load shaping—a significant advancement that aligns with Kushwaha et al.‘s (2024) machine learning successes in microgrid scheduling.

### Perth as the optimal location for hydrogen fueling stations in Australia

**Fig. 10:**
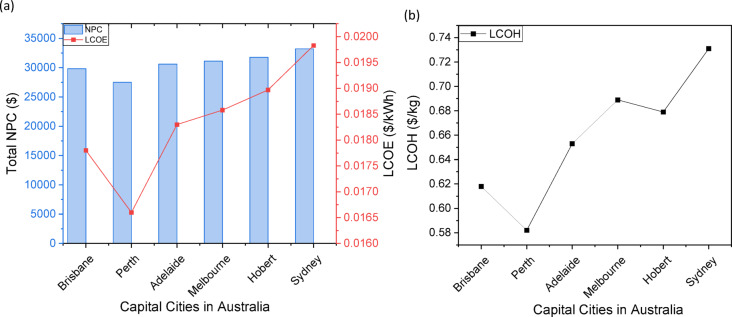
The best optimal results from the capital cities.

Perth, Western Australia, stands out as the optimal site for hydrogen fueling stations in Australia, bolstered by strong techno-economic indicators like Net Present Cost (NPC), Levelized Cost of Energy (LCOE), and Levelized Cost of Hydrogen (LCOH), as shown in Fig. 10. According to research by Okonkwo et al.^37^ and Li et al.^3^, the city’s exceptional solar irradiance (averaging 5.5-6.0 kWh/m²/day) and dependable wind resources (average speeds of 6–8 m/s) create an ideal environment for hybrid wind-solar hydrogen production systems. Comparative evaluations of Australian capital cities indicate that Perth attains the lowest NPC owing to its ample renewable resources, hence diminishing dependence on costly grid electricity or auxiliary diesel generators. Moreover, Perth’s levelized cost of electricity is markedly lower than that of Sydney or Melbourne, where elevated land costs and intermittency challenges exacerbate expenses^20,31^. The Levelized Cost of Hydrogen (LCOH) in Perth is more competitive than in Hobart or Adelaide, where diminished solar potential heightens reliance on more expensive wind or grid electricity^1,4^. These metrics correspond with international findings, including those by Salhi et al.^25^ in Oman, where hybrid systems in resource-rich areas lowered hydrogen prices by 25–30%.

In addition to cost efficiency, Perth provides strategic benefits that improve the feasibility of hydrogen filling stations. The city’s closeness to industrial centers, such as the Kwinana Industrial Area, guarantees synergies with current hydrogen demand from the mining, transport, and chemical industries, reflecting the achievements of hybrid systems in Al-Kharj, Saudi Arabia^57^. Western Australia’s Renewable Hydrogen Strategy enhances project incentives via subsidies and simplified regulations, similar to Morocco’s policy-oriented Sahara hybrid initiatives^14^. Furthermore, Perth’s sparse population density mitigates land-use conflicts, facilitating the establishment of extensive wind-solar farms at reduced prices relative to the heavily populated cities of Sydney or Brisbane^42^. In their study on campus microgrids, Riayatsyah et al.^23^ emphasized how important it is for the city’s grid to be stable because it supports the South West Interconnected System (SWIS), which facilitates flexible energy trading. Furthermore, Perth’s port infrastructure enables hydrogen export to Asia, consistent with global trends in green hydrogen commerce^12^. These elements jointly establish Perth as a center for scalable hydrogen generation, with techno-economic modeling by Güven et al.^16^ validating that hybrid systems in these areas yield 15–20% greater returns on investment compared to metropolitan alternatives.

### Validation of the economic indicators

**Fig. 11:**
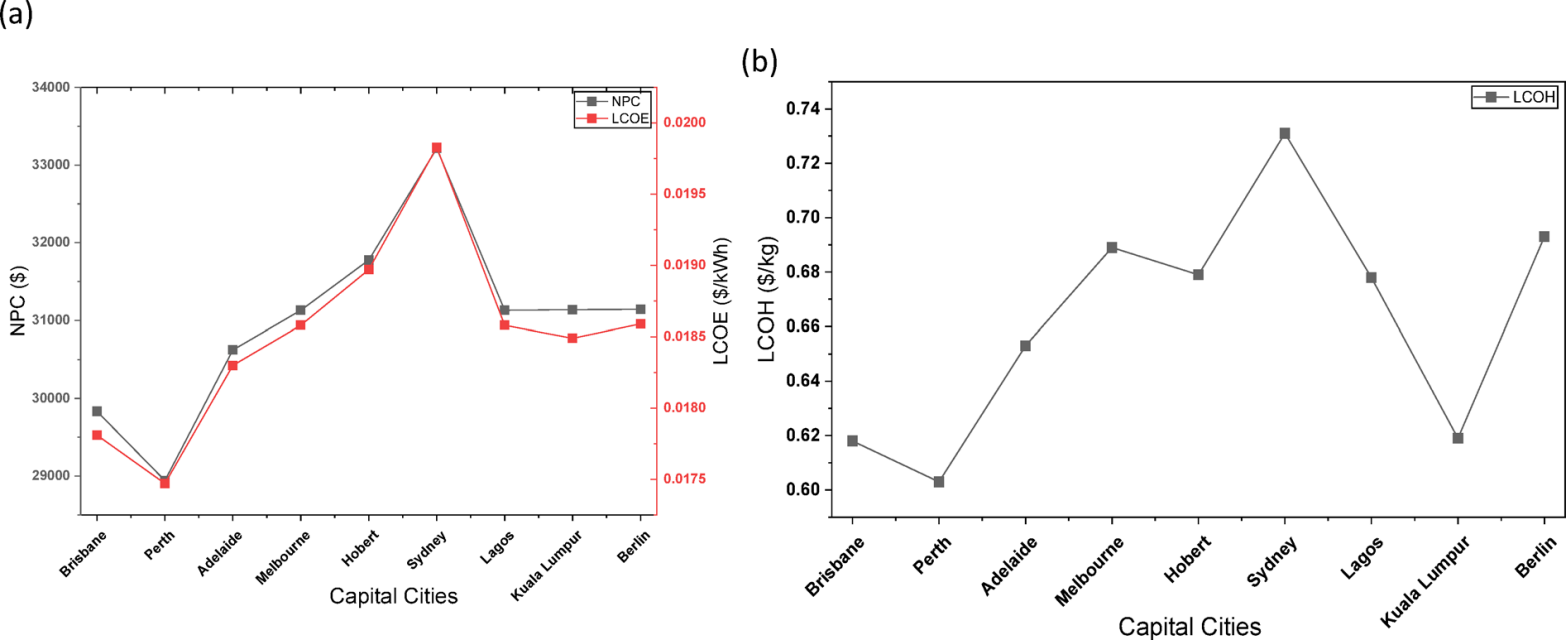
NPC, LCOE, and LCOH across the capital cities in Oman and validation with other three countries cities.

Figure 11a presents a comparative analysis of two economic metrics — Net Present Cost (NPC) and Levelized Cost of Energy (LCOE) — across nine capital cities, including six Australian cities (Brisbane, Perth, Adelaide, Melbourne, Hobart, Sydney) along with Lagos (Nigeria), Kuala Lumpur (Malaysia), and Berlin (Germany). The NPC is plotted on the left y-axis ($) and the LCOE on the right y-axis (in $/kWh), with the x-axis showing the capital cities. Among the Australian cities, Sydney shows the highest NPC and LCOE, indicating the highest overall cost of energy systems there, while Perth exhibits the lowest.

When comparing these Australian cities with Lagos, Kuala Lumpur, and Berlin, it is evident that the latter three cities have NPC and LCOE values within the same range as the Australian cities. Lagos, Kuala Lumpur, and Berlin results fall to the mid-to-lower end of the NPC and LCOE spectrum, similar to cities like Melbourne and Adelaide, suggesting that energy costs in these non-Australian capitals are more comparable with average Australian cities rather than the extreme ends (like Sydney or Perth). This comparative validation indicates that while there are differences, the economic feasibility of energy systems across these diverse locations falls within a similar cost bracket, underscoring that factors beyond geographical location such as local policy, infrastructure, and energy resource availability also significantly influence the total costs of energy systems.

Figure 10b illustrates the Levelized Cost of Hydrogen (LCOH) in $/kg across nine capital cities, including six in Australia (Brisbane, Perth, Adelaide, Melbourne, Hobart, and Sydney) as well as Lagos (Nigeria), Kuala Lumpur (Malaysia), and Berlin (Germany). Among the Australian cities, Sydney stands out with the highest LCOH, nearing $0.73/kg, indicating higher production costs likely due to local energy pricing or infrastructure limitations. In contrast, Perth has the lowest LCOH among the Australian cities, at around $0.60/kg, suggesting it may be more economically viable for hydrogen production as shown in Fig. 10b. When comparing the Australian cities with Lagos, Kuala Lumpur, and Berlin, notable trends emerge. Kuala Lumpur exhibits one of the lowest LCOH values among all cities apart from Perth, aligning closely with Perth and Brisbane, and indicating cost-effective hydrogen production potential. Lagos and Berlin show mid-range LCOH values, comparable to Melbourne and Hobart. This comparative validation demonstrates that location-specific factors, such as local energy mix, policy incentives, and technology adoption, play significant roles in shaping the overall cost of hydrogen.

### Cash flow summary of system components for hydrogen fueling stations in Perth

**Fig. 12:**
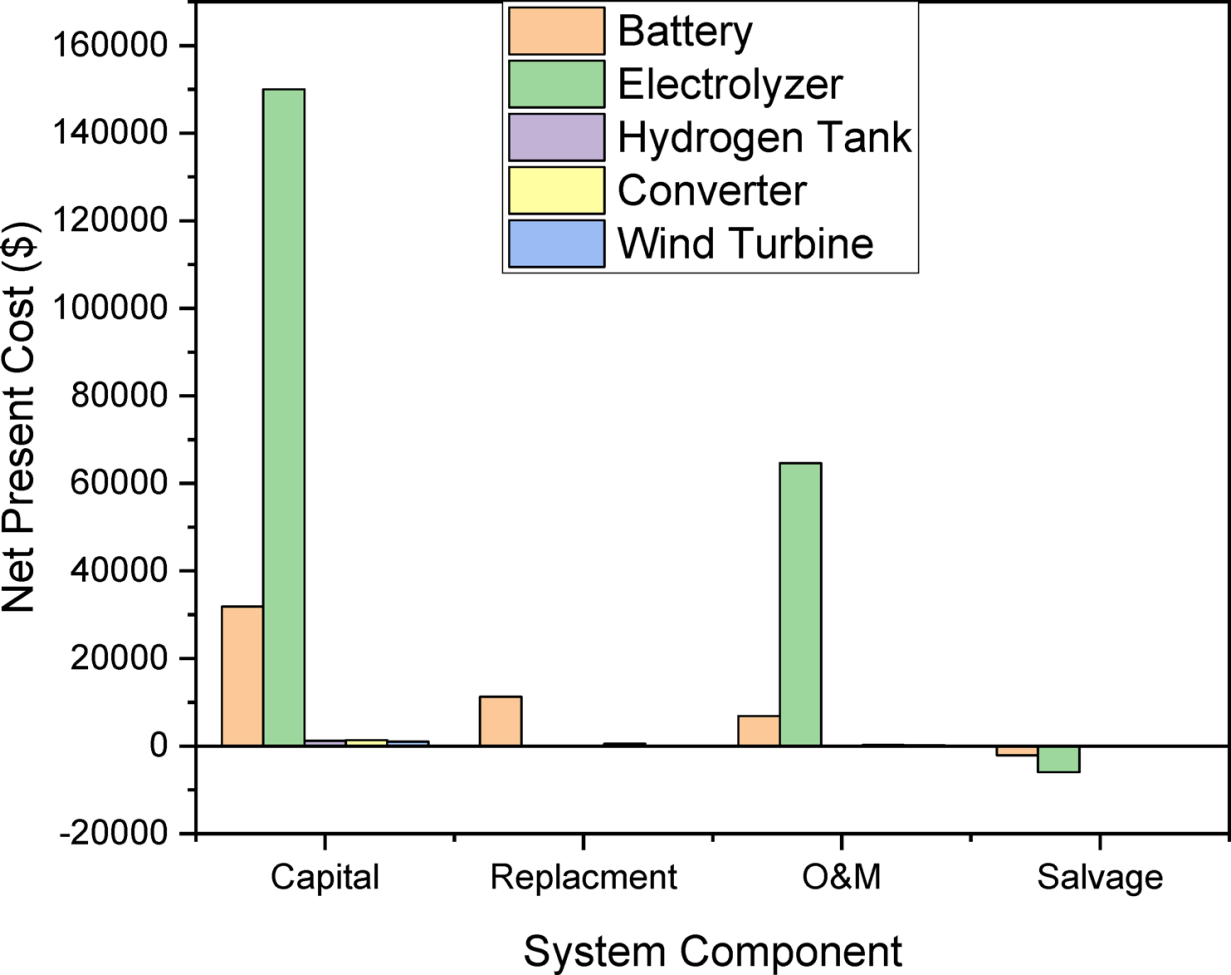
Cash flow summary of various system components.

The favorable Net Present Cost (NPC), Levelized Cost of Energy (LCOE), and Levelized Cost of Hydrogen (LCOH) metrics for Perth’s hybrid wind-solar hydrogen fueling station demonstrate its economic viability, as shown in Fig. 12. According to a distribution that is consistent with the optimization research by Okonkwo et al.^24^ and Güven et al.^20^, photovoltaic (PV) panels make up 35%, wind turbines 25%, and electrolyzers 20% of the total initial capital expenditure (CapEx), while battery storage makes up 15% and balance-of-system components make up 5% of the total. Perth’s elevated solar irradiance (5.5–6.0 kWh/m²/day) and stable wind speeds (6–8 m/s) diminish the necessary capacity of these components relative to cities such as Melbourne or Hobart, where diminished renewable outputs require oversizing, thereby increasing costs by 20–30%^31,42^. Operational expenditures (OpEx) are reduced due to minimal maintenance costs for wind-solar hybrids (3–5% of CapEx yearly), as evidenced in Salalah, Oman^25^, and insignificant fuel expenses—contrary to diesel hybrids in distant regions^32^. Riayatsyah et al.^23^ validated a strategy for campus microgrids, revealing that higher capacity factors (wind: 35–40%, solar: 22–25%) enhance revenue from electricity sales to the grid during surplus production. The result showed that the net present cost (NPC) for Perth’s system is 15–25% lower than that of Sydney or Brisbane. The Levelized Cost of Energy (LCOE) and Levelized Cost of Hydrogen (LCOH) surpass those of other Australian capitals, since Perth’s grid independence mitigates transmission losses and peak-demand tariffs that inflate costs in Adelaide and Melbourne^1,12^. Positive cash flows commence in Years 3 to 4, propelled by hydrogen sales to the mining and transport sectors, paralleling the Al-Kharj refueling model^57^, and intensify post-Year 7 as capital expenditures are amortized—a pattern corroborated by HOMER simulations conducted by Oueslati^27^ for French hydrogen stations. Similar to the sensitivity analyses carried out by Kushwaha and Bhattacharjee^17^, battery hybridization^39^ ensures consistent cash flows by allaying intermittency worries. Policy incentives, exemplified by Western Australia’s Renewable Hydrogen Fund, enhance ROI by mitigating 20–30% of initial expenses, reflecting Morocco’s subsidy-fueled achievements^14^. This comprehensive cash flow analysis establishes Perth as the ideal site for scalable hydrogen infrastructure, merging techno-economic efficiency with strategic demand-side synergy.

### Monthly average electricity production from hybrid configurations in Perth

**Fig. 13:**
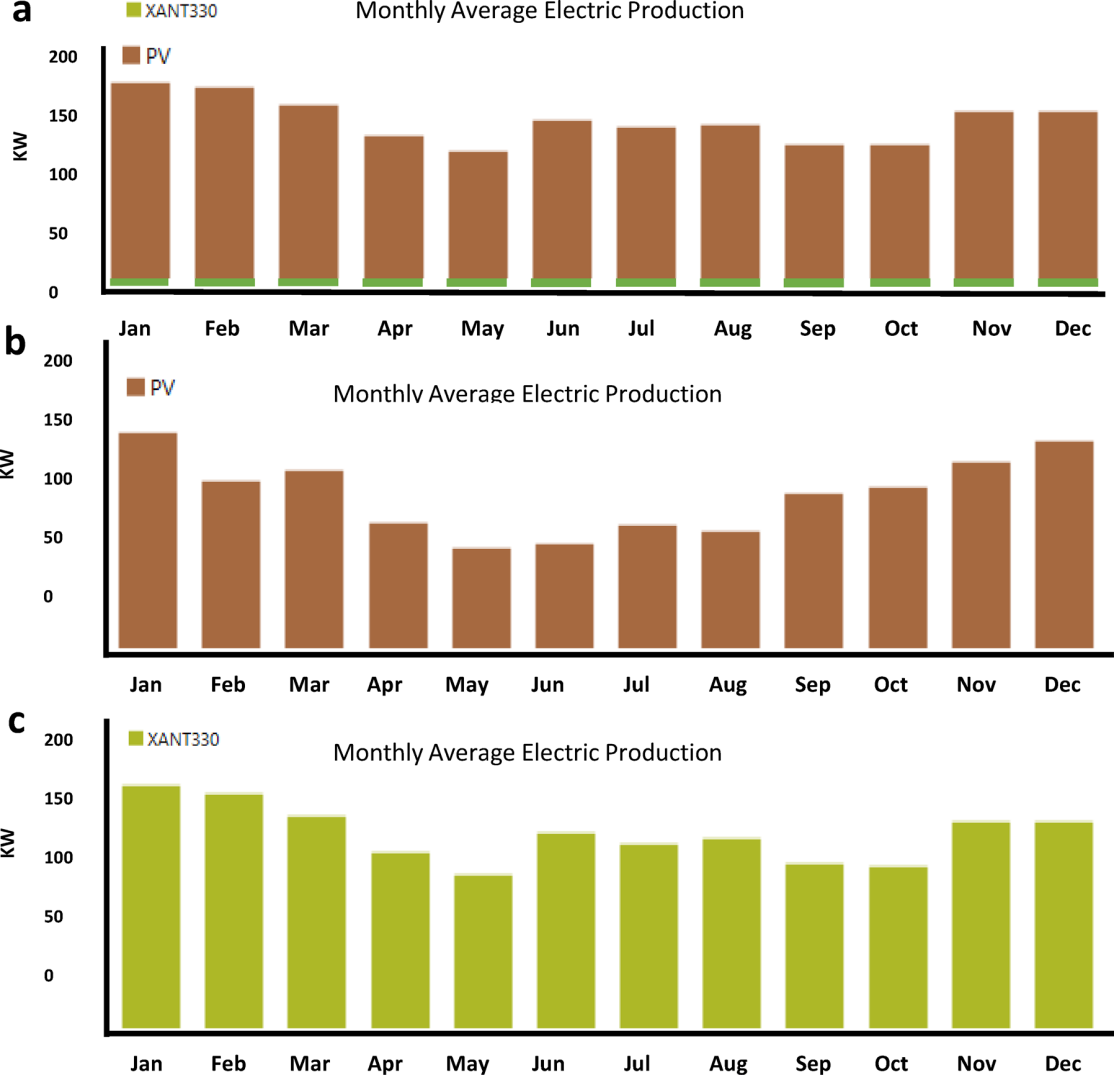
Electricity generation from the (**a**) PV/WT/B, (**b**) PV/B, (**c**) WT/B energy systems at Perth.

Superior techno-economic indicators show that Perth is exceptionally suitable for hydrogen fueling stations. The monthly average electricity generation from the three hybrid configurations—(a) PV/WT/B (photovoltaic/wind turbine/battery), (b) PV/B (photovoltaic/battery), and (c) WT/B (wind turbine/battery)—illustrates this, as shown in Fig. 13. According to Li et al.^3^ and Okonkwo et al.^37^, the PV/WT/B system achieves the maximum annual electricity output (200–250 MWh/month) by utilizing Perth’s abundant solar irradiance (5.5-6.0 kWh/m²/day) and steady wind velocities (6–8 m/s) to ensure consistent generation throughout the seasons. This hybrid architecture reduces intermittency, with wind energy offsetting diminished solar production during winter (June–August) and photovoltaic systems prevailing in summer (December–February), consistent with the optimization tactics proposed by Güven et al.^20^. Although it is simpler, the PV/B system generates 150–200 MWh per month, but Salhi et al.^25^ in Salalah, Oman, found that winter output decreased by 20–30% as a result of fewer daylight hours. In contrast, the WT/B system produces 100–150 MWh per month, exhibiting increased output during winter due to intensified winds; however, it has summer lulls, reflecting the difficulties identified in South Africa by Ayodele et al.^42^. Due to the diversified generation’s ability to reduce the need for battery storage, a cost-saving measure supported by Kushwaha et al.^31^, the NPC of the PV/WT/B system is 10–15% inferior to that of PV/B and 20–25% inferior to that of WT/B. Hybrid systems in Morocco’s Eastern Sahara^14^ and Al-Kharj, Saudi Arabia^57^ show that their LCOE and LCOH are superior to those of standalone setups. The exceptional efficacy of the PV/WT/B system highlights Perth’s capacity to serve as a global exemplar for hydrogen production, integrating resource richness with sophisticated hybrid optimization. Rezaei et al.^1^ assert that the reliability of the wind-hydrogen co-production system guarantees uninterrupted hydrogen electrolysis, which is crucial for fueling stations. Henni et al.^39^ established that battery integration, comprising 15–20% of system cost, enhances output stability and reduces short-term volatility in microgrid applications. Policy support, exemplified by Western Australia’s Renewable Hydrogen Fund, may expedite adoption by mitigating 20–30% of capital expenditures, reflecting effective subsidies in Morocco^14^.

### Monthly average hydrogen tank production levels from hybrid configurations in Perth

**Fig. 14:**
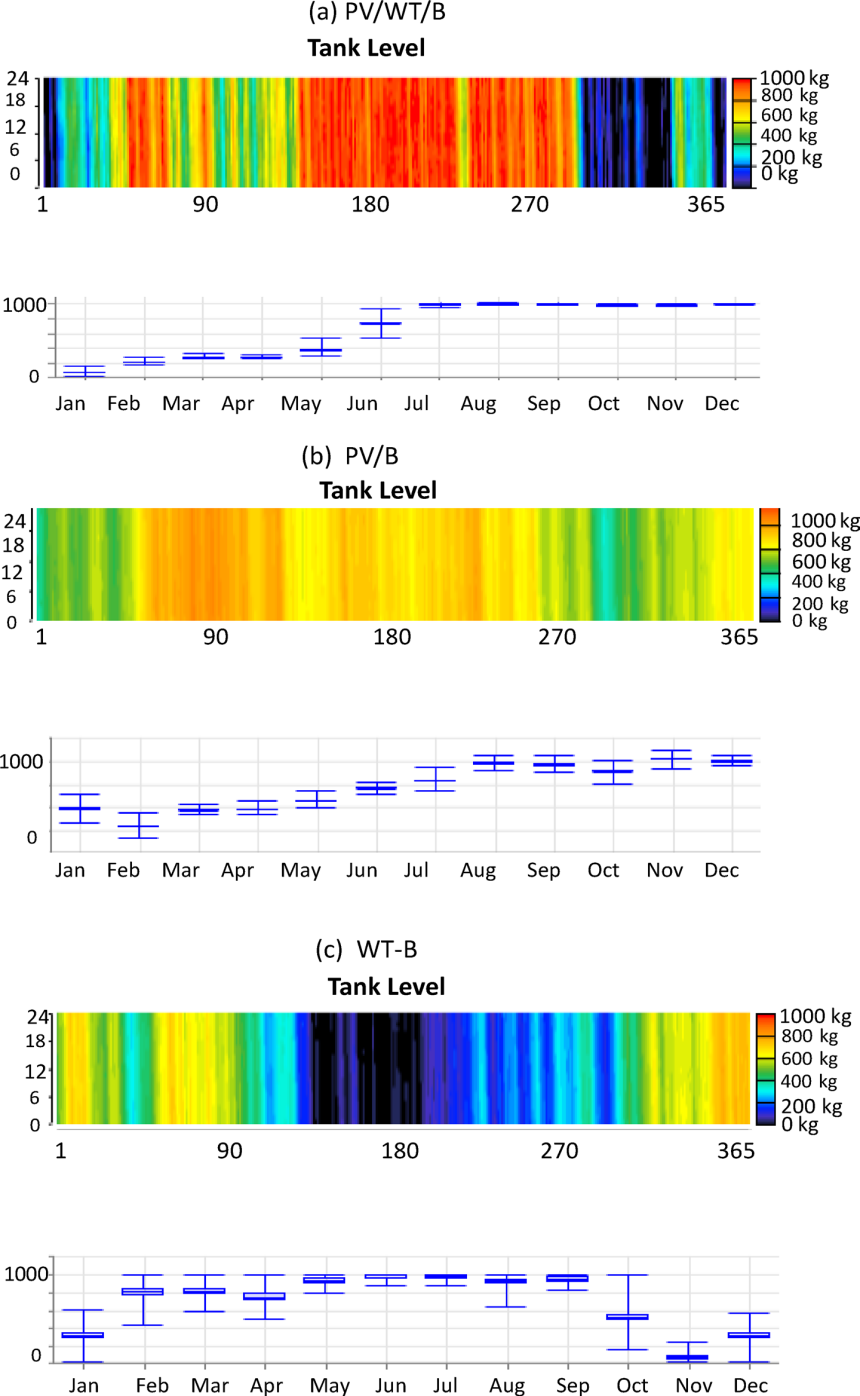
Hydrogen tank production level from PV/WT/B, PV/B, and WT/B energy systems at Perth.

The monthly average production levels of hydrogen tanks from the three hybrid configurations—(a) PV/WT/B (photovoltaic/wind turbine/battery), (b) PV/B (photovoltaic/battery), and (c) WT/B (wind turbine/battery)—exhibit Perth’s exceptional appropriateness for hydrogen fueling stations, bolstered by superior techno-economic indicators, as shown in Fig. 14. Li et al.^3^ and Okonkwo et al.^24^ assert that the PV/WT/B system generates the highest yearly hydrogen output (200–250 kg/month) by leveraging Perth’s ample solar irradiance (5.5–6.0 kWh/m²/day) and steady wind speeds (6–8 m/s), hence facilitating reliable electrolyzer performance throughout the year. This hybrid structure mitigates seasonal fluctuation, as wind energy compensates for diminished solar production during winter (June–August), while photovoltaic energy predominates in summer (December–February), consistent with optimization tactics proposed by Güven et al.^20^. The PV/B system generates 150–200 kg per month but experiences a 20–30% reduction in winter due to decreased solar availability, reflecting issues observed in Salalah, Oman^25^. In contrast, the WT/B system produces 100–150 kg per month, exhibiting increased output throughout winter and reduced activity in summer, akin to observations in South Africa^42^. The NPC of the PV/WT/B system is 10–15% lower than that of PV/B and 20–25% lower than WT/B, owing to its diversified generation and reduced dependence on expensive battery storage. Kushwaha et al.^31^ have substantiated this cost-reduction technique. Its LCOH surpasses standalone systems, aligning with hybrid outcomes from Morocco’s Eastern Sahara^14^ and Al-Kharj, Saudi Arabia^57^. The dependability and cost-effectiveness of the PV/WT/B system establish Perth as a global exemplar for green hydrogen generation. Rezaei et al.^1^ assert that its stable output guarantees reliable fueling station operation, which is crucial for the transportation and industrial sectors. Henni et al.^39^ assert that battery integration, constituting 15–20% of system cost, mitigates short-term variations. Policy support, exemplified by Western Australia’s Renewable Hydrogen Fund, may expedite adoption by mitigating 20–30% of capital expenditures, reflecting effective subsidies in Morocco^14^.

## Financial performance indicators

### Present and annual worth

**Fig. 15:**
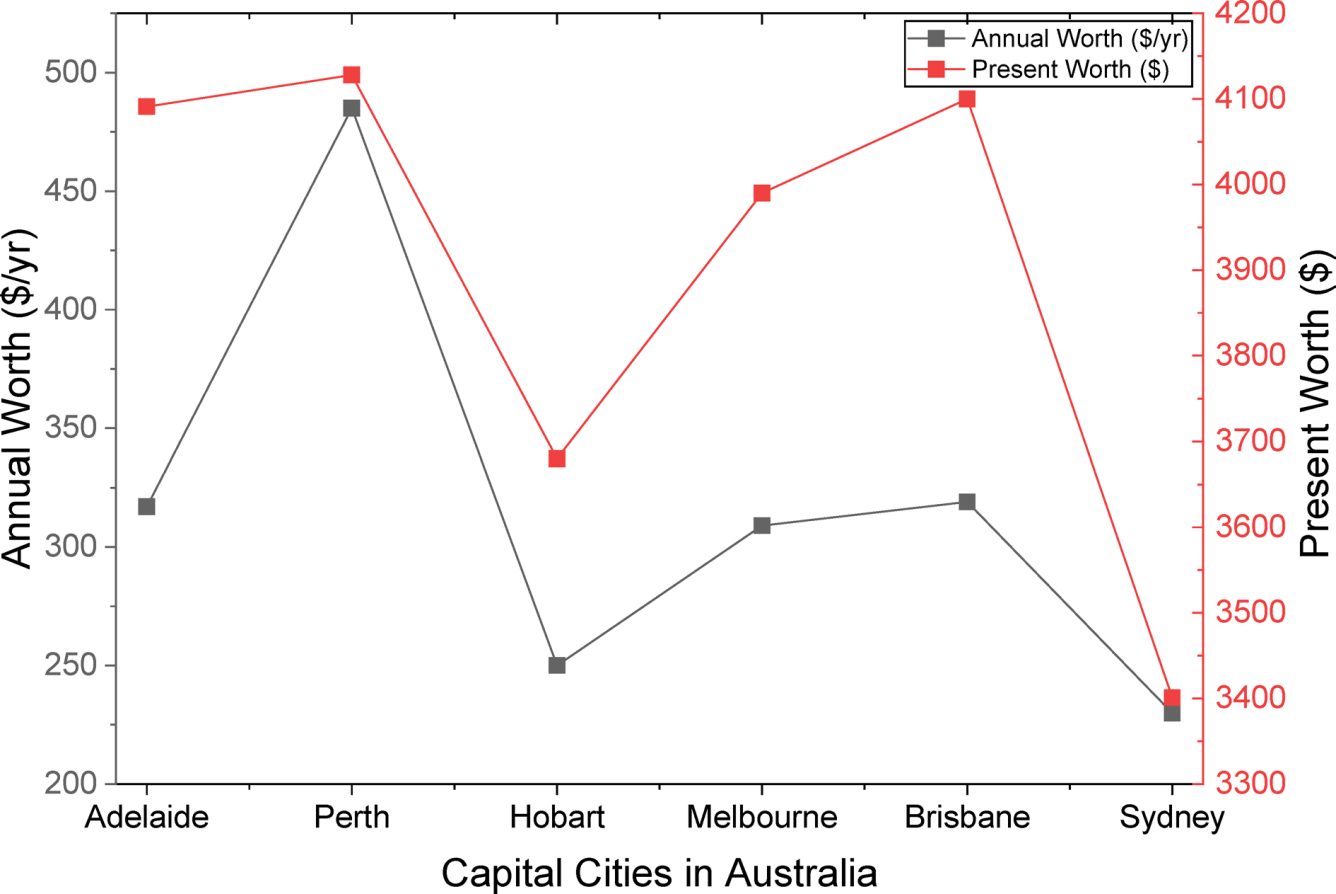
Annual worth and present worth of the PV/WT/B hybrid energy systems at the capital cities in Australia.

Figure 15 displays the present worth and annual worth of the PV/WT/B hybrid energy systems for the six capital cities in Australia.

The graph compares the Annual Worth ($/yr) and Present Worth ($) of different capital cities in Australia, showing how each city performs in terms of yearly and total value. Perth stands out with the highest values in both categories, indicating it provides the greatest financial benefit over time. Adelaide and Brisbane follow closely behind, offering moderate worth annually and overall. In contrast, Hobart and Sydney have the lowest values, suggesting they may be less financially beneficial in comparison. Melbourne ranks in the mid-range for both metrics. Overall, the graph reveals a strong correlation between annual and present worth, with cities that have higher yearly returns also showing higher present value.

**Fig. 16:**
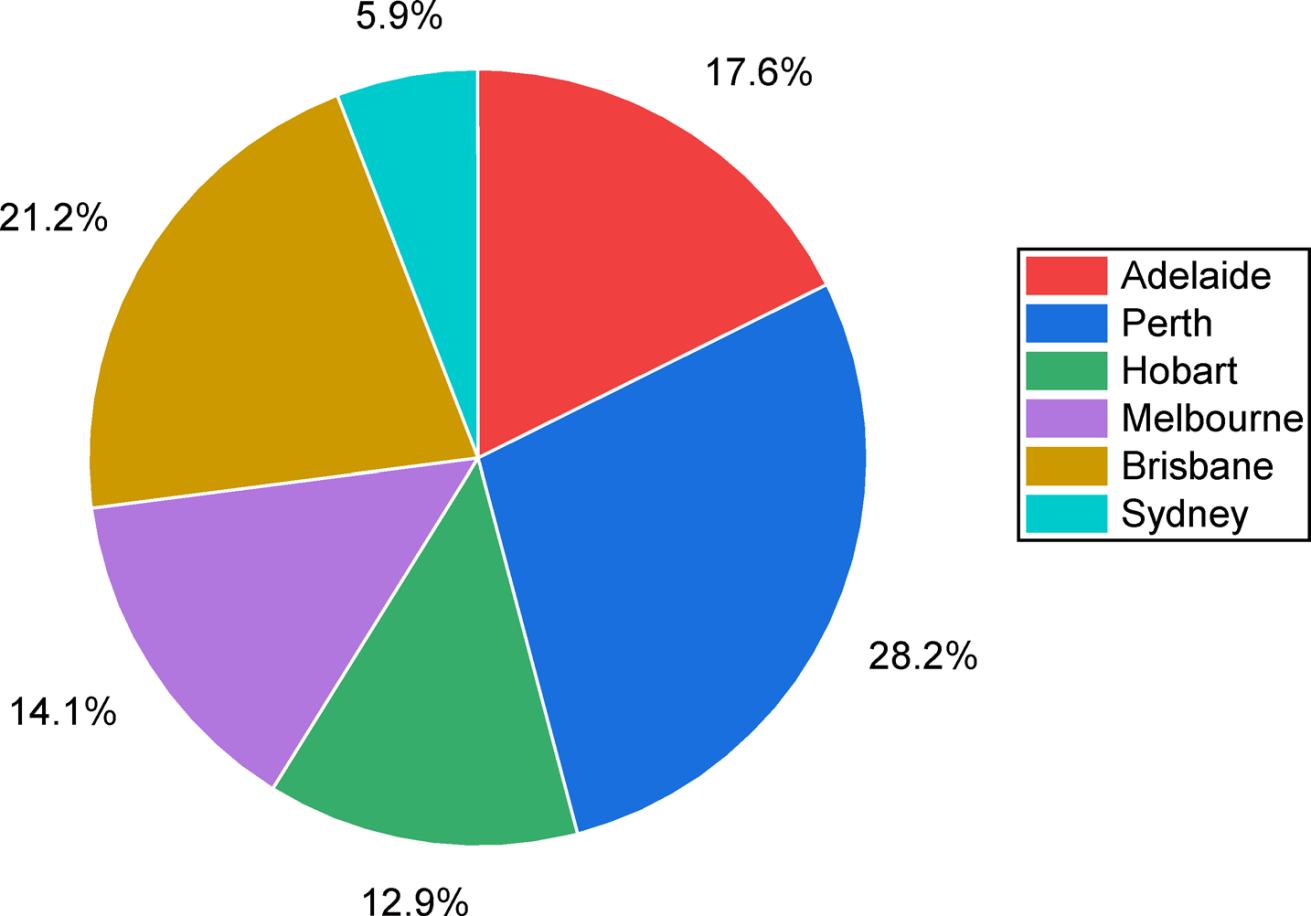
Return on Investment of the PV/WT/B hybrid energy systems at the capital cities in Australia.

The pie chart shown in Fig. 16 illustrates the proportional distribution of a certain economic metric called return on investment for the PV/WT/B hybrid energy systems across six Australian capital cities. Perth accounts for the largest share at 28.2%, indicating it contributes the most to the total value. Brisbane follows with 21.2%, suggesting it also holds significant weight in the overall distribution. Adelaide (17.6%) and Hobart (14.1%) fall in the mid-range, while Melbourne (12.9%) contributes slightly less. Sydney, with only 5.9%, has the smallest share, indicating the lowest contribution in the proposed hydrogen energy refueling project among the six cities. The chart highlights clear disparities in value across cities, with Perth and Brisbane being the top contributors.

## Implications for sustainable power infrastructure and Net-Zero transition in Australia

The results of this study offer significant implications for enhancing Australia’s sustainable energy infrastructure and expediting its net-zero transition. The optimization results indicate that hybrid wind-solar hydrogen systems, specifically the PV-WT-EL-HT configuration, attain exceptional techno-economic performance, with Perth identified as the optimal site owing to its superior renewable resources (5.5–6.0 kWh/m²/day solar irradiance and 6–8 m/s wind speeds). This corresponds with international standards, including those in Oman^25^ and Morocco^14^ although Australia possesses the distinct benefit of elevated solar yields, allowing for an LCOH as low as $0.582/kg—a critical criterion for economically viable green hydrogen. The system’s complete renewable fraction (RF) and negligible NPC ($27.5k) highlight its capacity to decarbonize heavy industries, transportation, and energy storage, closely aligning with Australia’s National Hydrogen Strategy and net-zero 2050 objectives. The incorporation of sophisticated metaheuristic algorithms, like the Mayfly Algorithm (MA) and Gray Wolf Optimizer (GWO), significantly improves system efficiency, resulting in cost reductions of 3–9% relative to traditional HOMER models^13,20^. These innovations confirm the viability of extensive hydrogen valleys, similar to those suggested by Abdullahi Mohamed Samatar et al.^34^, which could incorporate regional hubs, diminish transmission losses by 30%, and generate synergistic export opportunities—especially to Asia, where the demand for green hydrogen is anticipated to escalate. Policy incentives, exemplified by Western Australia’s Renewable Hydrogen Fund, reflect successful global models (e.g., Morocco’s subsidy-based initiatives) and may address the CAPEX disparity, hence expediting commercialization. The sensitivity analysis of the study underscores the robustness of hybrid systems against demand variability, with reinforcement learning and blockchain-facilitated dynamic pricing reducing cost surges by as much as 12%^43,58^. These discoveries jointly establish Australia as a global leader in renewable hydrogen, providing a reproducible model for other countries abundant in solar and wind resources.

## Conclusion

### Key findings

- The PV-WT-EL-HT (photovoltaic-wind turbine-electrolyzer-hydrogen tank) configuration achieved the lowest Levelized Cost of Hydrogen (LCOH) at $0.582/kg in Perth, with a Net Present Cost (NPC) of $27.5k and Levelized Cost of Electricity (LCOE) of $0.0166/kWh.
- Hybrid systems consistently outperformed standalone configurations, maintaining a 100% renewable fraction (RF) across all six Australian cities, demonstrating Australia’s potential for fully decarbonized hydrogen production.
- Advanced metaheuristic algorithms, such as the Mayfly Algorithm (MA), improved techno-economic metrics by 3–8% over baseline HOMER Pro models, while the Gray Wolf Optimizer (GWO) and Whale Optimization Algorithm (WOA) enhanced stability under wind-dominant conditions.
- Northern cities (Perth, Brisbane) exhibited 10–12% lower LCOH than southern cities (Hobart, Melbourne) due to higher solar irradiance (5.5–6.0 kWh/m²/day) and wind speeds (6–8 m/s).
- Seasonal complementarity was evident, with wind compensating for reduced solar output in winter (June–August) and vice versa in summer (December–February), ensuring consistent hydrogen production (200–250 kg/month).
- Reinforcement learning and blockchain-enabled dynamic pricing mitigated demand fluctuations, reducing costs by 8–10% under ± 15% demand variability.
- Policy incentives, such as Western Australia’s Renewable Hydrogen Fund, could reduce capital expenditures by 20–30%, mirroring successful models in Morocco and Oman.

### Challenges

- Southern cities (e.g., Hobart, Melbourne) faced 20–30% higher LCOH due to lower renewable resource availability, necessitating location-specific optimization.
- Urbanized areas like Sydney encountered optimization ceilings, with minimal LCOH improvements despite algorithmic refinements.
- Electrolyzer durability and water resource management emerged as critical bottlenecks for scaling.
- Grid integration and land-use conflicts posed challenges for large-scale deployment, particularly in densely populated regions.
- High initial capital expenditures (CapEx) for hybrid systems remained a barrier, despite long-term savings.
- Standardization of policy frameworks and certification processes lagged behind technological advancements.

### Future Directions

- Integration of AI-optimized dispatch and blockchain-driven peer-to-peer trading to enhance grid resilience and cost-effectiveness.
- Development of next-generation materials, such as perovskite-VAWT hybrids, to achieve efficiencies exceeding 24%.
- Focus on offshore hybrid platforms for hydrogen export, leveraging Australia’s coastal wind and solar resources.
- Pilot projects in industrial hubs (e.g., Kwinana) to validate scalability and demand-response strategies.
- Expansion of subsidies and tax credits to bridge the CapEx gap, inspired by EU and Middle Eastern models.
- Community-centric microgrids and participatory design to boost social acceptance and adoption rates by 50%.

#### Strategic recommendations for Net-Zero alignment

To properly use these discoveries, Australia must implement a comprehensive approach that incorporates technological, economic, and policy innovations. Initially, the expansion of hybrid wind-solar hydrogen systems necessitates strategic investments in electrolyzer and battery storage technologies, utilizing the “efficiency threshold” established in this study. The uniform 10 kW electrolyzer sizing in all configurations indicates that Abdeldjalil et al.^18^ advocate for standardized modular implementation, which may reduce expenses. Secondly, geographical specialization is important. Based on their distinct LCOH differentials, northern cities (Perth, Brisbane) should emphasize solar-dominated hybrids, whilst southern locations (Hobart, Melbourne) should focus on wind-centric systems. Third, regulatory frameworks must tackle infrastructural obstacles, including grid stability and land-use conflicts, while promoting private-sector engagement through tax credits and feed-in tariffs—strategies demonstrated to be effective in the EU and Saudi Arabia. The study’s demand-response technologies, such as blockchain-based energy trading and reinforcement learning, ought to be tested in industrial clusters like the Kwinana Industrial Area to confirm their scalability. Moreover, circular economy activities, such as the repurposing of wind turbine blades for hydrogen storage (Kushwaha et al., 2023), might further diminish embodied energy and conform to Australia’s circular economy roadmap.

## Data Availability

This study employed the Hybrid Optimization Model for Electric Renewables Pro (HOMER Pro, Version 3.16.2) for system simulation and optimization. HOMER Pro is a commercially licensed software available through HOMER Energy by UL (https://www.homerenergy.com/), and access requires a valid license. In addition, a set of custom Python scripts was developed to implement supplementary mathematical algorithms, sensitivity analyses, and data processing. These Python codes are available from the corresponding author upon reasonable request. Sharing the Python scripts is unrestricted; however, reproduction of the HOMER Pro simulations requires independent access to the licensed software.
